# Dynamic Activity of miR-125b and miR-93 during Murine Neural Stem Cell Differentiation *In Vitro* and in the Subventricular Zone Neurogenic Niche

**DOI:** 10.1371/journal.pone.0067411

**Published:** 2013-06-27

**Authors:** Annalisa Lattanzi, Bernhard Gentner, Daniela Corno, Tiziano Di Tomaso, Pieter Mestdagh, Frank Speleman, Luigi Naldini, Angela Gritti

**Affiliations:** 1 San Raffaele Scientific Institute, Telethon Institute for Gene Therapy (TIGET), Milano, Italy; 2 Department of Experimental Medicine and Biochemical Sciences, Section of Biochemistry and Molecular Biology, University of Perugia, Perugia, Italy; 3 Center for Medical Genetics, Ghent University Hospital, Ghent, Belgium; 4 Vita - Salute San Raffaele University Medical School, Milano, Italy; University of South Florida, United States of America

## Abstract

Several microRNAs (miRNAs) that are either specifically enriched or highly expressed in neurons and glia have been described, but the identification of miRNAs modulating neural stem cell (NSC) biology remains elusive. In this study, we exploited high throughput miRNA expression profiling to identify candidate miRNAs enriched in NSC/early progenitors derived from the murine subventricular zone (SVZ). Then, we used lentiviral miRNA sensor vectors (LV.miRT) to monitor the activity of shortlisted miRNAs with cellular and temporal resolution during NSC differentiation, taking advantage of *in vitro* and *in vivo* models that recapitulate physiological neurogenesis and gliogenesis and using known neuronal- and glial-specific miRNAs as reference. The LV.miRT platform allowed us monitoring endogenous miRNA activity in low represented cell populations within a bulk culture or within the complexity of CNS tissue, with high sensitivity and specificity. In this way we validated and extended previous results on the neuronal-specific miR-124 and the astroglial-specific miR-23a. Importantly, we describe for the first time a cell type- and differentiation stage-specific modulation of miR-93 and miR-125b in SVZ-derived NSC cultures and in the SVZ neurogenic niche *in vivo*, suggesting key roles of these miRNAs in regulating NSC function.

## Introduction

MicroRNAs (miRNAs) are small non-coding-RNAs that regulate the expression of a large fraction of mRNA in a sequence-specific manner [1] through translational repression and/or transcript destabilization [2], [3]. The dramatic changes in expression levels of some miRNAs with CNS specific/enriched expression profiles [4], [5], [6], [7] suggest their critical role as regulators of the developmental pathways that contribute to the complexity and cellular diversity of the adult nervous system [8], [9].

Indeed, an increasing amount of studies address miRNA function in multiple steps of neurogenesis, including self-renewal and specification of neural stem cells (NSCs), migration and maturation of young neurons, and functional integration of mature neurons in the neural circuitry [10], [11]. Among the most widely studied, miR-124 is a neuronal fate determinant in cell cultures [12], [13] and in the subventricular zone (SVZ) neurogenic niche [14], [15], while miR-125b promotes neuronal differentiation and regulation of synaptic function [16], [17], [18]. However, the identification of individual miRNAs that might specifically identify NSCs and play a functional role in modulating NSC self-renewal and multipotency remains largely incomplete.

Functional studies of individual miRNAs in the CNS require techniques allowing to simultaneously monitor spatio-temporal expression patterns and cellular localization. To this end, strategies based on the visualization of miRNA-regulated reporters have been developed [8] that could overcome the low sensitivity of histology and *in situ* RNA expression [19], [20]. Recently, miRNA-regulation has been implemented in the context of lentivirally delivered transgenes. In lentiviral (LV) miRNA sensor vectors (LV.miRT) the expression of a reporter gene is regulated by perfectly matched miRNA target (T) sequences. The expression of the reporter gene is downregulated when the cognate miRNA is active within the cell [21]. LV.miRT allow segregating transgene expression between different CNS lineages (i.e. neurons versus astrocytes) [22], [15], separating out neural precursors in ES-derived pluripotent cultures [23] as well as selecting/maintaining human pluripotent cell populations in culture [24]. Thus, a similar strategy could possibly be used to enrich for NSCs or committed progenitors, providing large amounts of neural cells suitable for transplantation in different neurodegenerative pathologies. In this perspective, a comprehensive knowledge of the modulation of specific miRNAs during NSC maintenance/differentiation is needed.

Here, we used global miRNA expression profiling to identify candidate miRNAs enriched in NSC populations. Then, we applied the LV.miRT platform to monitor the activity of shortlisted miRNAs during NSC differentiation, exploiting several *in vitro* and *in vivo* experimental settings that recapitulate physiological neurogenesis and gliogenesis and using known neuronal- and glial-specific miRNAs as reference. We found that miR-93, a member of the miR-106b-25 cluster, and the brain-associated miR-125b are highly enriched in somatic NSCs, and their expression and activity are significantly modulated in NSC-derived progeny, with distinct temporal progression as well as lineage- and cell type-specific patterns of modulation. Furthermore, we highlighted a positive correlation between the expression of both miRNAs in NSCs and their proliferative activity.

Our studies validate “sensor” LVs as a sensitive tool to monitor the temporal patterns of endogenous miRNA activity at the cellular level. Also, they provide for the first time a comprehensive analysis of the dynamic activity of miR-93 and miR-125b during lineage commitment and differentiation of murine somatic NSCs in culture systems and in the SVZ stem cell niche during physiological neurogenesis.

## Materials and Methods

### Ethics Statement

All animals were handled in strict accordance with the ARRIVE guidelines. Protocols were approved by the Institutional Committee for the Good Animal Experimentation of the San Raffaele Scientific Institute (IACUC #420).

CD1 mice (adult males and pregnant females) were purchased by Charles River (Calco, LC, Italy) and housed in the SPF animal facility of the San Raffaele Scientific Institute.

### Transfer Vector Plasmids

We used monocystronic and bidirectional (bd) self-inactivating-LVs, the latter allowing the coordinate dual expression of two transgenes driven by the human phosphoglycerate kinase (PGK) promoter [21], [25]. *LV.CTRL* encodes for GFP, *bdLV.CTRL* encodes for two reporter genes (GFP and the monomeric (m) Cherry). MiRNA target sequences were cloned into the XbaI-XmaI site, downstream of the GFP marker gene of *(bd)LV.CTRL,* as previously described [21], [26]. Briefly, mature miRNA sequences (hsa-miR) were obtained from the miRNA registry (http://microrna.sanger.ac.uk), and oligonucleotides containing 4 repeats of the reverse complement of the miRNA sequence were synthesized and cloned into the LV or bdLV.

List of oligonucleotides used to generate transfer vector plasmids:

miR-125b sense 1: ctagatcacaagttagggtctcagggacgattcacaagttagggtctcagggaacgcgt.

miR-125b sense 2: tcacaagttagggtctcagggatcactcacaagttagggtctcagggac.

miR-125b antisense 1: tccctgagaccctaacttgtgaatcgtccctgagaccctaacttgtgat.

miR-125b antisense 2: ccgggtccctgagaccctaacttgtgagtgatccctgagaccctaacttgtgaacgcgt.

miR-124 sense 1: ctagataatggcattcaccgcgtgccttaattcgaatggcattcaccgcgtgccttaaacgcgt.

miR-124 sense 2: tggcattcaccgcgtgccttaaatgcattggcattcaccgcgtgccttaac.

miR-124 antisense 1: ttaaggcacgcggtgaatgccattcgaattaaggcacgcggtgaatgccattat.

miR-124 antisense 2: ccgggttaaggcacgcggtgaatgccaatgcatttaaggcacgcggtgaatgccaacgcgt.

miR-93-5p sense1: ctagactacctgcacgaacagcactttgttcgaactacctgcacgaacagcactttgacgcgt.

miR-93-5p sense2: ctacctgcacgaacagcactttgatgcatctacctgcacgaacagcactttgc.

miR-93-5p antisense1: caaagtgctgttcgtgcaggtagttcgaacaaagtgctgttcgtgcaggtagt.

miR-93-5p antisense2: ccgggcaaagtgctgttcgtgcaggtagatgcatcaaagtgctgttcgtgcaggtagacgcg.

miR-23a sense: ctagatagggaaatccctggcaatgtgatcgatggaaatccctggcaatgtgatc.

miR-23a antisense: ccgggatcacattgccagggatttccatcgatcacattgccagggatttccctat.

NB: 4 copies of miR-23a were generated by successive ligation of 2 oligonucleotide products (each containing 2 tandem repeats complementary to miR-23a) into the pBlueNA subcloning construct.

### Vector Production and Titration

Vector production and titration were performed as described previously [21], [25]. Briefly, VSV-pseudotyped third-generation LV were produced by transient four-plasmid co-transfection into 293T cells and purified by ultracentrifugation as described [25]. Vector titer was tested on 293T cells by limiting dilution and estimated by means of qPCR for HIV genome copies. Vector particles were measured by HIV-1 gag p24 antigen immunocapture (NEN Life Science Products, Waltham, MA, USA). Vector infectivity was calculated as the ratio between titer and particles. Details can be found in [25]. Vector titers were in the range between 2 to 5×10^9^ TU/ml (bdLVs) and 5 to 8×10^9^ TU/ml (monocystronic LVs). Infectivity was higher than 5×10^4^ TU/ng of p24 for all vectors.

### Cell Cultures

#### Neural stem cells

Post-natal day (PND) 2 CD1 mice were anaesthetised in crushed ice before being decapitated. Brains were removed and tissue containing the subventricular zone (SVZ) of the forebrain lateral ventricles was dissected. Tissues from 3 mice were pooled to establish and expand NSC cultures (n = 2–5 independent cultures) using the NeuroSphere Assay (NSA), as previously described [27]. For all the experiments we used serially passaged NSCs (passage 5th – 20th).

Establishment of NSC-derived populations at different stages of commitment/differentiation was performed as previously described [27] and summarized in Figure S1. Briefly, serially passaged neurospheres were dissociated and grown for 24 hours in serum-free DMEM/F12 (1∶1 vol:vol) containing insulin, apo-transferrin, putrescine and progesterone (*control medium*) containing FGF2 and EGF (*growth medium*), in order to obtain a population enriched in proliferating, undifferentiated cells (*stem/precursors; 1day, d*). This population was plated in the presence of an adhesion substrate, in control medium supplemented with FGF2 in order to obtain a population enriched in neuronal and glial committed progenitors at different stages of commitment (*progenitors; 3d*). Progenitors were exposed to control medium containing 10 ng/ml leukaemia inhibitory factor (LIF) or 2% FBS and grown for additional 1d, 4d and 7d (*differentiated cells*) in order to achieve cultures at progressive stages of neuronal and glial differentiation/maturation. We used nestin to identify stem/precursor cells and immature glial precursor cells, glial fibrillary protein (GFAP) to identify astrocytes, β-tubulin III (βtubIII) to identify immature neurons, microtubule-associated protein 2 (Map2) and neuronal nuclear antigen (NeuN) to identify mature neurons.

#### SH-SY5Y neuroblastoma cells

The SH-SY5Y cell line (kindly provided by Dr. J. Meldolesi, San Raffaele Scientific Institute; originally purchased from ATTC, CRL-2266) was grown in DMEM/F12 (1∶1 vol:vol) supplemented with 10% FBS. Differentiation of SH-SY5Y cells was performed by plating 10^4^ cells/cm^2^ in DMEM/F12 containing 2% FBS and 10 µM retinoic acid for 3 days, followed by 4 days in the presence of serum-free DMEM/F12 supplemented with brain derived neurotrophic factor (BDNF; 10 ng/ml).

### Lentiviral-mediated Gene Transfer in NSCs

*Stem/precursor cells* were incubated overnight with LV or bdLV (2×10^7^ TU/ml; MOI 100). Medium was then removed and fresh medium added in order to obtain the formation of neurospheres, which were then subcultured every 4–5 days by mechanical dissociation. In this way we established stably transduced NSC lines that were further differentiated according to the protocol described above. We evaluated the efficacy of LV and bdLV transduction by quantifying: 1) the vector copy number (VCN) by qPCR; 2) the number of mCherry^+^ cells and GFP^+^ cells by FACS and immunohistochemistry.

The effect of bdLV transduction on NSC functional features (self-renewal, proliferation and differentiation capacity) was evaluated as previously described [27].

### Fluorescence Activated Cell Sorting (FACS)

Transduced cells were grown for at least 4 passages before undergoing FACS analysis, in order to reach steady-state mCherry and GFP expression and to rule out pseudo-transduction. Before FACS analysis, either free floating cells were collected or adherent cells were detached with Accumax (Sigma-Aldrich, St. Louis, MO, USA), washed, resuspended in PBS and analyzed by multi-color flow cytometry on a FACS Canto flow cytometer (Becton-Dickinson, San Jose, CA, USA). 7-Aminoctinomycin D (7-AAD; Sigma-Aldrich) was used to exclude dead cells. Transduced cells were identified by gating on 7-AAD^−/^mCherry^+^ cells. Direct GFP fluorescence was then analyzed.

MiRNA activity is expressed as fold repression (FR) of GFP expression measured in bdLV.miRT-transduced cells as compared to bdLV.CTRL-transduced cells. For this analysis we considered only the population expressing bright mCherry signal (mCherry^hi^). Transgene ratio (TGR) and FR was calculated as previously described [26]: FR = TGR_miRT_/TGR_CTRL_; TGR = MFI_GFP_/MFI_mCherry_.

### Intracerebral Delivery of Vectors

#### Adult mice

Two month-old CD1 mice were anesthetized with Avertine (Sigma-Aldrich). A hole was drilled in the skull and vectors (2×10^6^ TU/1.5 µl) were slowly injected (0.3 µl/min) unilaterally in the striatum by means of a 33G needle-Hamilton syringe. The needle was left in place for additional 3 min and then slowly withdrawn. Stereotactic coordinates in mm from Bregma (according to the Paxinos mouse brain atlas) were: AP +0.5, ML +2, DV −2.5.

#### Neonates

PND1 CD1 mice were anaesthetised in crushed ice and placed on a refrigerated stage. The head was trans-illuminated in order to identify the ventricles. Vectors (2×10^6^ TU/1.5 µl) were rapidly injected in the left lateral ventricle through a glass capillary mounted on a micromanipulator without exposing the skull. The procedure takes 3 to 5 minutes, the survival rate is >90%.

Forty days post-injection mice were deeply anesthetized with Avertine and intracardially perfused with 0.9% NaCl followed by ice-cold 4% PFA in PBS. Brains tissues were collected, equilibrated for 24 hours in 30% sucrose in PBS and included in agarose. Serial coronal vibratom-cut sections (6 series, 40 µm-thick) were processed for immunofluorescence analysis.

### Immunofluorescence Analysis

Immunofluorescence on cell cultures and tissue slices was performed as previously described [27], [28].

#### Primary antibodies

*R*abbit polyclonal anti-DS red (mCherry) (Clontech, Mountain View, CA, USA; 1∶500); mouse monoclonal anti-β-tubulin III (Babco, Richmond, CA, USA; 1∶1.000); rabbit polyclonal anti-β-tubulin III (Babco; 1∶500); mouse monoclonal anti-GFAP (Chemicon-Millipore, Temecula, CA, USA; 1∶1.000); rabbit polyclonal anti-GFAP (Dako, Glostrup, Denmark; 1∶1.000); mouse monoclonal anti-Map2 (Immunological Science, Rome, Italy; 1∶300); mouse monoclonal anti-nestin (Chemicon; 1∶200); mouse monoclonal anti-NeuN (Chemicon; 1∶500); rabbit polyclonal anti-Ki67 (Novocastra-Leica Biosystems GmbH, Nussloch, Germany; 1∶1.000); chicken polyclonal anti-GFP (Abcam, Cambridge, UK; 1∶1.000).

#### Secondary antibodies

Alexa 488-, Alexa 546- or Alexa 633-conjugated anti-mouse or anti rabbit IgG (1∶1.000, 1∶2.000 and 1∶500, respectively) (Molecular Probes; Carlsbad, CA, USA); Cy3- conjugated goat anti-mouse or goat anti-rabbit IgG (Jackson ImmunoResearch Laboratories, West Grove, PA, USA; 1∶500).

Coverslips and tissue sections were counterstained with dapi (4', 6-diamidino-2-phenylindole; Roche) or TO-PRO-3 (Life Technologies-Invitrogen, Carlsbad, CA, USA), washed in PBS, and mounted on glass slides using Fluorsave (Calbiochem-EMD Millipore, Billerica, MA, USA).

### Image Acquisition

Samples were visualized with: 1) Zeiss Axioskop2 microscope using double laser confocal microscopy with Zeiss Plan-Neofluar objective lens (Zeiss, Arese, Italy). Images were acquired using a Radiance 2100 camera (Bio-Rad, Segrate, Italy) and LaserSharp 2000 acquisition software (Bio-Rad); 2) Perkin Elmer UltraVIEW ERS Spinning Disk Confocal (PerkinElmer Life Sciences Inc., MA, USA); 3) Leica TCS SP2 three-laser confocal microscope with Leica Confocal Software (LCS; Leica Microsystems GmbH, Wetzlar, Germany).

### Cell Quantification

#### Cell cultures

We analyzed 3–4 coverslips for each antigen (>1000 cells) in each experiment, performing 2–3 independent experiments.

#### Tissue slices

We analyzed coronal brain sections (2–3 slices/region/mice; n = 3 mice/treatment group) selected within the striatal region (adult injection) or all along the SVZ-RMS-OB pathway (neonatal injection). For the cell type composition, data are expressed as percentage of immunoreactive (IR) cells (for each specific marker) on total nuclei (untreated mice), or on total transduced cells (GFP^+^ for LV.CTRL; mCherry^+^ for bdLVs). The OB was divided into different cell layers based on nuclear staining, including the glomerular layer (GlL), the external plexiform layer (ExPL), the internal plexiform layer (InPL), the mitral cell layer (MiL), the granule cell layer (GCL) and the medulla (Me). For each OB section, the number of transduced cells in each layer was expressed as the percentage on the total number of transduced cells (GFP^+^ for monocystronic LVs; mCherry^+^ for bdLVs).

For the quantification of miRT-mediated GFP expression, a total of 300–3000 cells were examined in each experimental group for GFP expression and co-localization with lineage-specific markers. Data are expressed as: i) percentage of marker^+^GFP^+^ cells (direct GFP fluorescence) on total number of marker^+^ cells (monocystronic LVs); ii) percentage of marker^+^GFP^+^ cells (direct GFP fluorescence) on total number of marker^+^mCherry^+^ cells (anti-mCherry antibody).

### Detection of LV Genome

Detection of LV genome was performed as previously described [28]. Genomic DNA was extracted from cell pellets (Maxwell, Promega, Madison, WI, USA) and quantified at NanoDrop ND-1000 Spectrophotometer (Euroclone, Pero, Italy). Vector copies per genome were quantified by TaqMan analysis starting from 100 ng of template DNA. Quantitative PCR was performed by amplifying the PSI sequence of the LV backbone using primers as follows: forward, 5′-TGAAAGCGAAAGGGAAACCA-3′, 750 nmol final concentration; reverse, 5′-CCGTGCGCGCTTCAG-3′, 200 nmol final concentration. PCR product length was 64 bp. The probe was 5′-VIC-AGCTCTCTCGACGCAGGACTCGGC- MGB-3′, 200 nmol final concentration. As internal reference for normalization, we amplified a fragment of the murine β-actin gene: forward, 5′-AGAGGGAAATCGTGCGTGAC-3′, 300 nmol final concentration; reverse, 5′-CAATAGTGATGACCTGGCCGT-3′, 750 nmol final concentration; probe, 5′-VIC-CACTGCCGCATCCTCTTCCTCCCMGB-3′, 200 nmol final concentration.

A standard curve of genomic DNA carrying 4 LV copies, validated by Southern blot analysis, was constructed using DNA extracted from transgenic mouse tissue. The standard curve, based on different dilutions of DNA (from 200 to 25 ng), and accordingly, of LV copies, was used as standard both for LVs and for β-actin amplification. Reactions were carried out in a total volume of 25 µl, in an ABI Prism 7900 HT Sequence Detection System (Life Technologies-Applied Biosystems, Carlsbad, CA, USA). The number of LV copies was calculated as follows: (ng LVs/ng endogenous DNA)×(number of LV integrations in the standard curve).

### Quantitative RT-PCR

Total RNA was isolated from *stem/precursor*, *progenitors* and *differentiated cells* using the miRNeasy Mini kit (Qiagen, Hilden, Germany) according to the manufacturer instructions. 200 ng of total RNA were reverse transcribed using High Capacity cDNA Reverse Transcription kit and specific miRNA primers (Life Technologies-Applied Biosystems, Carlsbad, CA, USA). TaqMan quantitative real-time PCR was performed with hsa-miR-16, hsa-let7a, hsa-miR-125b, mmu-miR93, mmu-miR-124a, hsa-miR-23a, hsa-miR-106b, hsa-miR-25 and hsa-miR-9 specific probes (Life Technologies-Applied Biosystems) on ABI7900 thermal cycler. Data were normalized on miR-9 expression level and the fold change to stem/precursors values was calculated trough ΔΔCt method.

### miRNA Expression Profiling

Murine mature miRNA expression levels were quantified using the stem-loop RT-qPCR platform (Life Technologies-Applied Biosystems). Briefly, 60 ng of total RNA was reverse transcribed using the rodent stem-loop RT Megaplex primer pools A and B (v2.0) followed by a 12-cycle pre-amplification according to the manufacturer’s instructions. Pre-amplified cDNA was diluted 1/4 and quantified using miRNA specific Taqman assays (Life Technologies-Applied Biosystems) in a 3.5 µl qPCR reaction containing 1.5 µl of Taqman assay (1/17 dilution of 20× solution), 1.75 µl Taqman gene expression master mix, 0.02 µl of cDNA and 0.23 µl of water on a 7900 HT qPCR system (Life Technologies-Applied Biosystems). A proper normalization strategy is a crucial aspect of the RT-qPCR data analysis workflow. For large-scale miRNA expression profiling studies it has been previously shown that mean expression value normalization outperforms the normalization strategies that make use of small RNA controls. For this reason, raw miRNA expression values were filtered using a Cq-cutoff of 32 and normalized using the global mean, as previously described [29], [30].

### Statistics

Data were analyzed with Graph Pad Prism version 5.0a for Macintosh. Unpaired Student t-test, One-Way or Two-Way analysis of variance followed by post-tests were used according to data sets and p≤0.05 was considered to be statistically significant. The number of samples analyzed and the statistical test used are indicated in the figure legends.

## Results

### miRNA Expression Profile during NSC Differentiation

**Figure 1:**
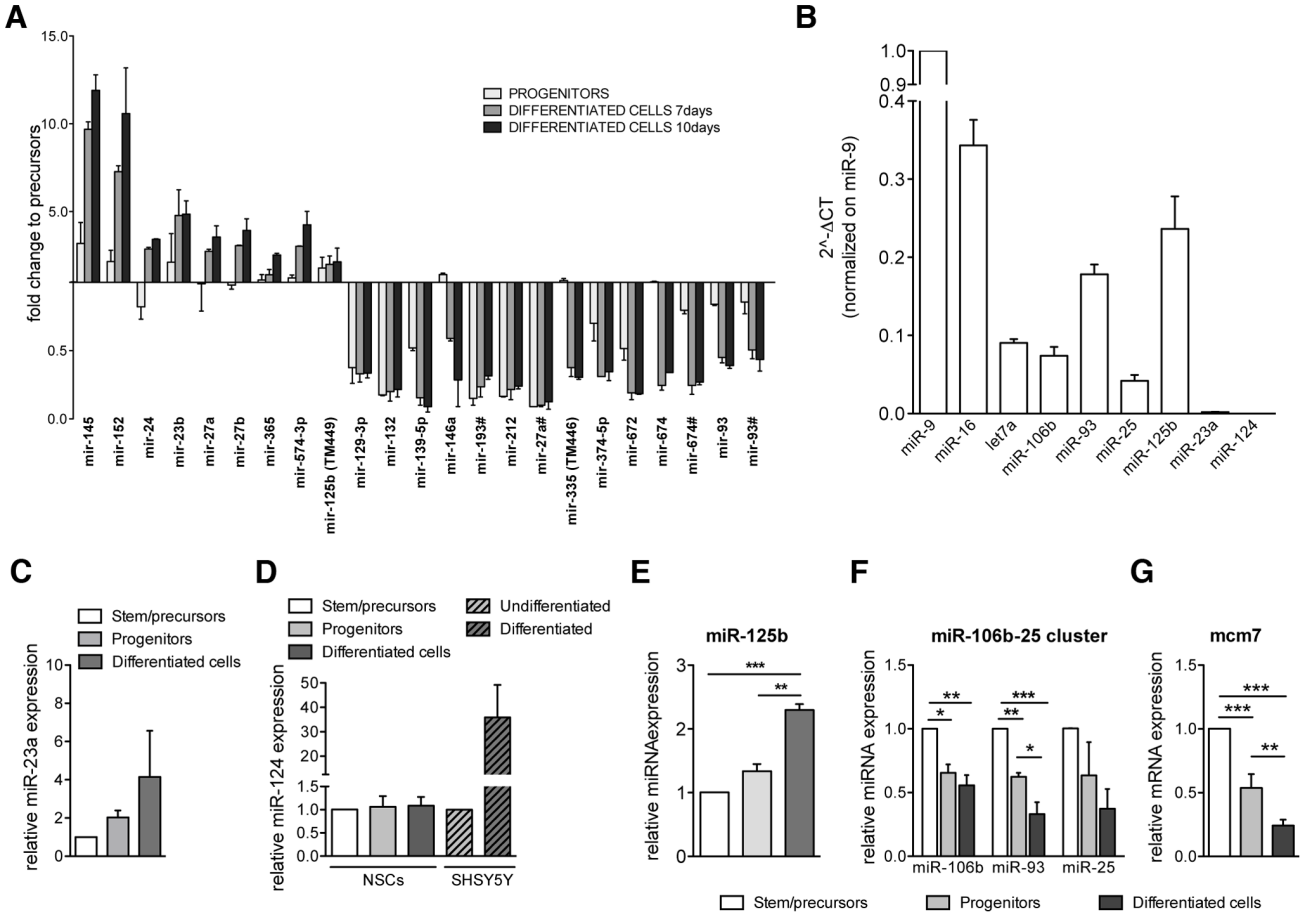
Modulation of miRNA expression in NSCs during differentiation. (**A**) Top ranked miRNAs from genome-wide expression profiling showing ≥2 fold-change in expression in *progenitors* and *differentiated cells* (7 and 10 days in vitro) when compared to *stem/precursors*. (**B**) Relative abundance of miRNAs in *stem/precursors*. Expression levels are normalized on miR-9 and plotted as 2^∧−ΔCt^ values, according to qRT-PCR expression data. Mean ± SEM; n = 3–4 independent NSC lines in triplicate. (**C**) Expression levels of miR-23a in *progenitors* and *differentiated cells* relative to *stem/precursors*. Mean ± SEM; n = 4 independent NSC lines in triplicate. (**D**) Expression levels of miR-124 in *progenitors* and *differentiated cells* relative to *stem/precursors* (NSCs) compared to levels in SH-SY5Y neuroblastoma cells before (undifferentiated) or after exposure to retinoic acid/BDNF (differentiated). Data are mean ± SEM; n = 3 independent experiments with 4 independent NSC lines. (**E**) Modulation of miR-125b expression during NSC differentiation. Data are expressed as fold to *stem/precursors*. Mean ± SEM, n = 4 independent experiments, n = 5 independent NSC lines. (**F**) Relative expression levels of miR-106b, miR-93 and miR-25 in *progenitors* and *differentiated cells* as compared to *stem/precursors*. Mean ± SEM; n = 2 independent experiments, n = 3 independent NSC lines. (**G**) Relative expression of mcm7 mRNA in *progenitors* and *differentiated cells* as compared to *stem/precursors*. Mean ± SEM; n = 3 independent experiments, n = 5 independent NSC lines. (E, F, G): One way analysis of variance followed by Bonferroni’s posttest.*p<0.05; **p<0.01; ***p<0.001.

We isolated and cultured NSCs from the murine neonatal subventricular zone (SVZ) [31], [27] and established NSC-derived populations at progressive stages of commitment/differentiation, identified as *stem/precursors*, *committed progenitors* and *differentiated cells* (7d and 10d *in vitro*) [32] (**Figure S1**). On these NSC-derived populations we performed a high-throughput miRNA RT-qPCR analysis [30] in order to identify miRNAs specifically expressed and/or significantly modulated upon NSC differentiation. By using stem-loop RT-qPCR platform we interrogated 535 mammalian miRNAs. Among them, 201 displayed detectable expression level. In Table S1 we report the full list of miRNAs analyzed and their expression levels (expressed as ΔCt). From this large dataset we shortlisted 33 miRNAs displaying modulation as a function of lineage commitment and differentiation, on the base of differential expression (ΔΔCt ≥1) in *progenitors* and/or *differentiated cells* as compared to *stem/precursors* (**Table S2**). We further selected 9 and 14 miRNAs that were significantly upregulated and downregulated, respectively (**Figure 1A**).

Among the upregulated miRNAs there were some expected candidates, such as the miR-145-152 cluster [24] and the miR-24-23-27 cluster, previously reported to be upregulated during differentiation of neural progenitors in the astroglial lineage [33], [34]. A moderate upregulation of expression levels in differentiated cultures was observed for the brain-associated miR-125b. This miRNA has been described to promote neuronal differentiation and synaptic function [16], [35], [18]. However, the involvement of miR-125b in the regulation of cell proliferation and apoptosis [36], and the indication that nestin is a direct functional target of miR-125b [37] suggest a possible implication of this miRNA in NSC homeostasis. These data, and the hypothesis that the weak upregulation of miRNA expression observed in bulk populations might overestimate cell subsets with differential miRNA expression/activity, prompted us to consider miR-125b as an interesting candidate to be further investigated in our NSC culture model.

Among the downregulated miRNAs we found the miR-132/212 cluster, which is involved in CNS development and embryonic stem (ES) cell biology [38], [39], [40] and miR-93, which belongs to the miR106b-25 cluster located on murine chromosome 5, in the 13th intron of the host gene mcm7 [41]. This miRNA cluster has been implicated in the regulation of neural progenitor cell proliferation and neuronal differentiation [42] but the potential role of miR-93 in modulating somatic NSC function is still elusive.

Based on the available data and on our expression profile we selected miR-93 and miR-125b for further analysis, in order to assess their activity in stem/early progenitor cells and the cell-specific modulation during lineage commitment and differentiation, considering the neuronal-specific miR-124 [11], [43] and the astroglial-specific miR-23a [33] as reference.

Quantitative PCR analysis confirmed that miR-125b and miR-93 are abundantly expressed in *stem/precursor cells* when compared to miR-23a and miR-124 (**Figure 1B**). Expression of miR-23a was upregulated in *differentiated cells* as compared to *stem/precursors*, as expected (**Figure 1C**). Surprisingly, this trend was not observed for miR-124 (**Figure 1D**). This apparent discrepancy was likely due to the low percentage of mature neurons in NSC-derived differentiated cultures (10–20%; see Figure S1) when compared to homogeneous neuronal cultures (i.e. neurons from SH-SY5Y neuroblastoma cells) (**Figure 1D**). While miR-125b expression increased upon cell commitment and was maintained at high level in the differentiated progeny (**Figure 1E**), miR-93 was significantly downregulated during NSC differentiation (**Figure 1F**). We observed a similar trend of expression of miR-106b and miR-25, the other two miRNAs in the cluster (**Figure 1F**) even though their absolute expression is lower with respect to miR-93 (see **Figure 1B**). Also, expression levels of these miRNAs during NSC differentiation paralleled those of the host gene mcm7 (**Figure 1G**), strongly suggesting that they are co-transcribed in the context of the mcm7 primary transcript.

While these data suggested a potential role for miR-93 and miR-125b in the maintenance of stem/early progenitor cells and/or in lineage commitment, they also highlighted the limitation of qPCR-based analysis in detecting modulation of miRNA-expression in low-represented subset of cells within a bulk culture. This prompted us to investigate the activity of the selected miRNAs at the cell level using a novel LV-based reporter system.

### Modulation of miR-124 and miR-23a Activity in NSC-derived Neurons and Astroglia

**Figure 2:**
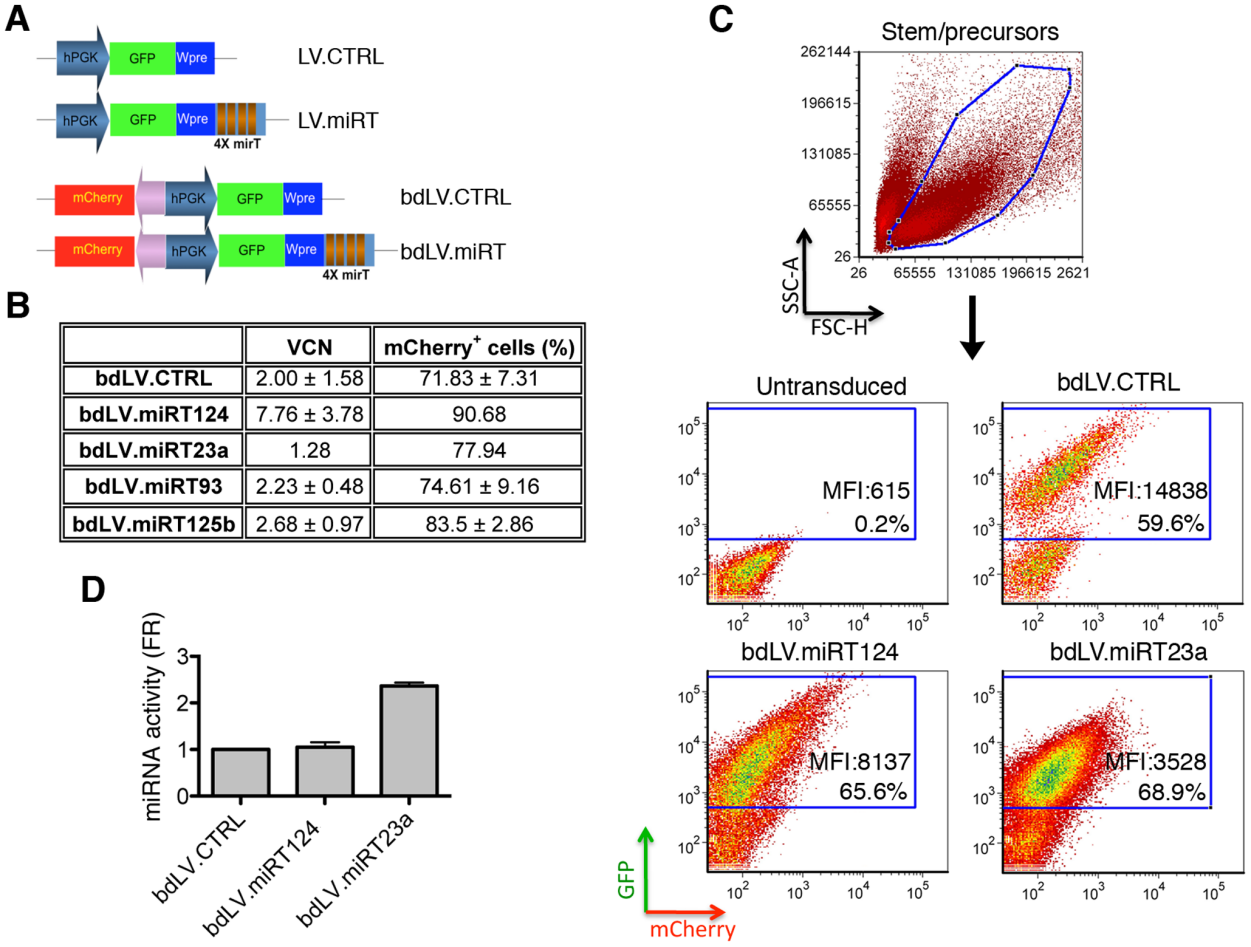
miR-124 and miR-23a activity in NSCs monitored using LV.mirT. (**A**) Cartoon showing monocystronic (LVs) and bidirectional (bdLV) miRNA-regulated (LV.miRT, bdLV.miRT) and control vectors (LV.CTRL, bdLV.CTRL). (**B**) Efficacy of transduction in NSCs measured as VCN (assessed by taqman qPCR) and as percentage of mCherry^+^ cells (assessed by indirect immunofluorescence). Data are mean ± SEM, n = 1–3 independent experiments. (**C**) Representative dot plots of untransduced, bdLV.CTRL- and bdLV.miRT-transduced ste*m/precursors* (bdLV.miRT23a and bdLV.miRT124) gated on physical parameters. GFP/mCherry expression is shown, the percentages in the plots indicate transduction levels. MFI, mean fluorescence intensity of GFP signal. (**D**) miRNA activity is expressed as fold repression (FR) of GFP expression measured in bdLV.miRT124- and bdLV.miRT23a-transduced cells as compared to bdLV.CTRL-transduced cells. FR was calculated as previously described [26]: FR = TGR_miRT_/TGR_CTRL_; TGR (transgene ratio) = MFI_GFP_/MFI_mCherry_. Data are the mean ± SEM; n = 3 independent experiments.

Lentiviral vectors (LV) expressing a reporter gene regulated by perfectly matched miRNA target sequences (miRT) can be exploited to dynamically monitor miRNA activity [44]. When miRNA expression reaches a threshold for activity in a cell, it binds the miRT in the vector-derived mRNA (in this case, a GFP reporter), efficiently inhibiting its expression [45], [21], [46]. In cells in which the miRNA is not expressed or falls below the activity threshold, GFP is proficiently expressed from the ubiquitous PGK promoter and can be detected by flow cytometry or direct immunofluorescence (IF) analysis. To reliably detect a negative GFP signal in cells with miRNA activity, we used a bidirectional (bd) LV.miRT co-expressing from the same PGK promoter a second fluorescence reporter (mCherry) not subjected to miRNA regulation (**Figure 2A**). In some experiments, we used monocystronic LV.miRT (**Figure 2A**), obtaining similar results. Vectors without miRNA-specific target sequences were used as controls (LV.CTRL, bdLV.CTRL; **Figure 2A**).

We transduced *stem/precursors* using bdLV.miRT23a, bdLV.miRT124 and bdLV.CTRL according to previously optimized protocols [28], [47]. Transduced cells were then expanded in culture as neurospheres for at least 3 passages before analysis. bdLV-transduced NSCs displayed between 1.3 and 7 vector copies integrated per genome (VCN), resulting in ≈70–90% of mCherry-immunoreactive (IR) cells (**Figure 2B**). Notably, transduction did not alter NSC long-term expansion, clonogenic efficiency and multipotency (**Figure S1**).

We then measured GFP and mCherry expression by FACS analysis in bdLV.CTRL- and bdLV.miRT-transduced cells (**Figure 2C**). The normalized suppression of GFP protein in bdLV.miRT-transduced cells correlates directly with the activity of endogenous miRNAs and was calculated as described previously [21], [48]. *Stem/precursors* transduced with bdLV.miRT124 and bdLV.CTRL expressed comparable levels of GFP after normalization to mCherry protein (fold repression = 1), thus indicating absence of miR-124 activity in this cell population. Interestingly, the 2-fold repression of GFP expression in bdLV.miRT23a-transduced *stem/precursor cells* suggested low basal activity of this miRNA in this population (**Figure 2D**).

**Figure 3:**
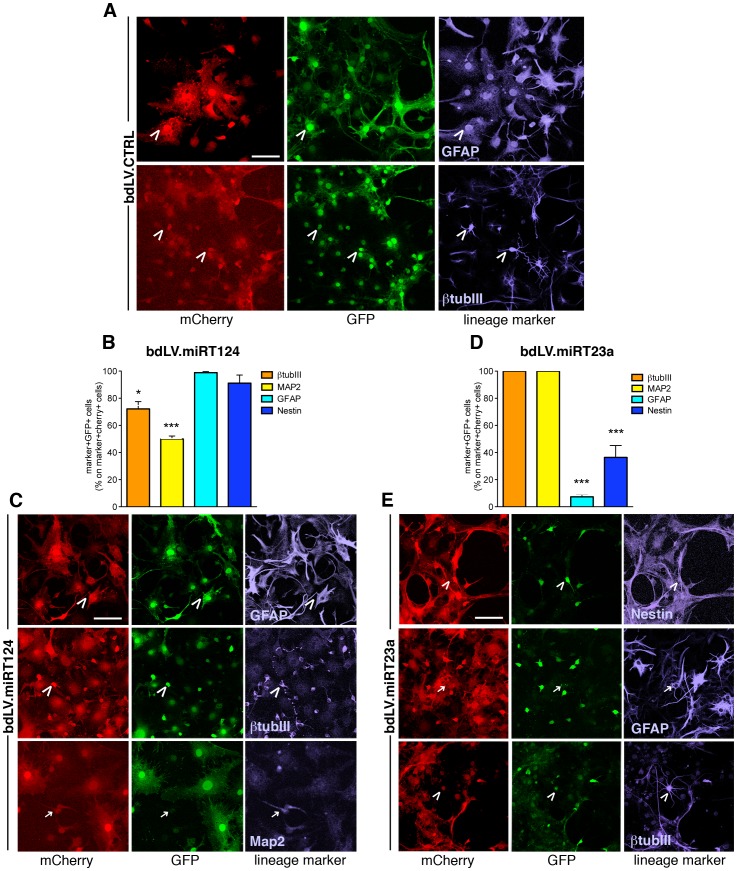
Activity of miR-124 and miR-23a in NSC-derived neurons and astrocytes. Qualitative and quantitative GFP expression in neurons (βtubIII, Map2; blue) and astroglial cells (GFAP, nestin; blue) in bdLV.CTRL-, bdLV.miRT124- and bdLV.miRT23a-transduced NSC-derived *differentiated cells* (10 days in vitro). (**A**) Transduced cells (red, anti-mCherry antibody) in bdLV.CTRL-transduced cultures express bright GFP (green; direct fluorescence). (B–E) A significant decrease of GFP expression is observed in bdLV.miRT124-transduced neurons (βtubIII, MAP2) (**B, C**) and in bdLV.miRT23a-transduced astrocytes (GFAP) and immature glial cells (nestin) (**D, E**). Arrowheads indicate GFP^+^marker^+^ (miR^−/low^) cells, arrows indicate GFP^−^marker^+^(miR^+/high^) cells. Data are the mean ± SEM; n = 3 experiments, 1–3 coverlips/antigen/experiment. Data for each marker in bdLV.miRT-transduced cells were compared to their counterpart in bdLV.CTRL-transduced cells using One-way analysis of variance followed by Bonferroni’s posttest. * p<0.05, *** p<0.001. Scale bars, 50 µm.

Cell counts following IF confocal analysis performed on the mCherry-expressing population in differentiated cultures (10d) indicated a miRT-mediated cell-type specific GFP repression in the NSC-derived differentiated progeny (**Figure 3**). In bdLV.CTRL-transduced cultures we detected 99.8%±0.12% (mean ± SEM; n = 5) of mCherry^+^GFP^+^ cells within the neuronal (βtubIII, Map2) and glial (Nestin, GFAP) populations, indicating transgene co-expression (**Figure 3A**). Robust downregulation of GFP expression in neurons derived from bdLV.miRT124-transduced NSCs (**Figure 3B, C**), and in glial cells derived from bdLV.miRT23a-transduced NSCs (**Figure 3D, E**) confirmed the upregulation of miR-124 and miR-23a during neuronal and glial differentiation, respectively.

Our results obtained using known neuronal- and glial-specific miRNAs validated the bdLV.miRT platform as a sensitive and specific tool to monitor endogenous miRNA activity at the single-cell level in low-represented subset of cells within mixed neuronal/glial cultures. Thus, we reasoned that this platform could be a powerful tool to monitor the activity of miRNAs that were shortlisted based on enriched expression in NSCs and modulation of expression during NSC differentiation.

### Cell-type Specific Modulation of miR-125b and miR-93 Activity during NSC Differentiation

In order to define a lineage- and/or functional-specific modulation of miR-93 and miR-125b activity in NSC-derived neuronal and glial progeny, we exploited the sensitivity and specificity of the bdLV.miRT platform in a time-course differentiation analysis.

We generated bdLV.miRT93 and bdLV.miRT125b and used them to transduce NSCs. BdLV.miRT-transduced NSC-derived populations were analyzed by FACS and by IF, using the same experimental protocol described for miR-23a and miR-124.

**Figure 4:**
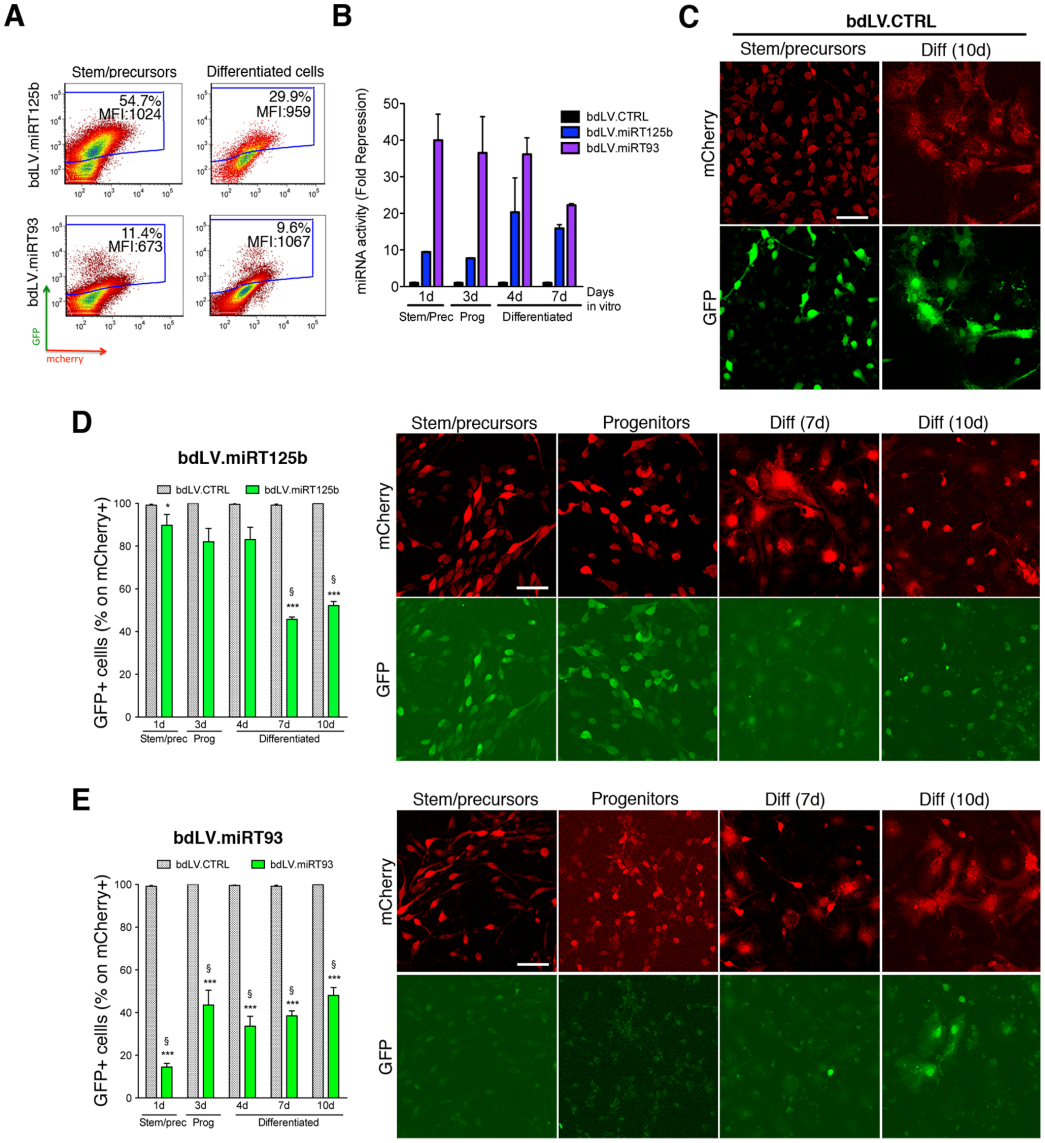
Modulation of miR-125b and miR-93 activity during NSC differentiation. (**A**) Representative GFP/mCherry expression in *stem/precursors* transduced with bdLV.miRT93 or bdLV.miRT125b (left), and their differentiated progeny (right). Percentages indicate GFP^+^ cells. MFI, mean fluorescence intensity of GFP signal. (**B**) Mean GFP fold repression (FR) measured in bdLV.miRT125b- and bdLV.miRT93-transduced cells as compared to bdLV.CTRL-transduced cells (mean ±SEM; n = 2 independent experiments). Calculations were done as in Figure 2D. (**C**) Co-expression of GFP and mCherry in bdLV.CTRL-transduced cells. Red, anti-mCherry antibody; green, GFP direct fluorescence. (**D**) Downregulation of GFP expression indicates increased activity of miR-125b at late stages of NSC differentiation in bdLV.miRT125b-transduced cells. (**E**) Faint GFP expression in bdLV.miRT93-transduced NSC cultures indicates robust activity of miR-93 in stem/precursor cells that is maintained in ≈50% of cells in progenitors and differentiated cells. Prec, *stem/precursors*; Prog, *progenitors*; Diff, *differentiated cells*; d, days in vitro. Data are expressed as percentage of GFP^+^ cells on transduced cells (mCherry^+^). Gray bars represent percentages in bdLV.CTRL-transduced cells. Data are the mean±SEM; n = 2 experiments, 3–5 coverlips/experiment. One-way analysis of variance followed by Bonferroni’s posttest. * p<0.05, *** p<0.001 versus bdLV.CTRL; § p<0.05 versus *stem/precursors.*

FACS analysis (**Figure 4A**) showed a 10-fold repression of GFP expression in bdLV.miRT125b-transduced *stem/precursors* and *progenitors*, which increased up to 20 fold after removal of FGF2 (**Figure 4A, B**), indicating upregulation of miR-125b activity at the beginning of lineage commitment that persisted in the differentiated populations. Interestingly, a 30–40-fold repression of GFP was measured in bdLV.miRT93-transduced *stem/precursors* and *progenitors,* indicating robust activity of miR-93, which significantly decreased upon cell differentiation (**Figure 4A, B**). FACS analysis of more differentiated neural cultures is challenging due to the difficulties in generating viable single cell suspensions. We therefore performed IF analysis followed by quantitative confocal microscopy to assess mCherry and GFP expression. The number of GFP^+^ cells (direct IF) was expressed as percentage on total mCherry^+^ cells (indirect IF). BdLV.CTRL-transduced cells co-expressed GFP and mCherry at all differentiation time points (**Figure 4C**; 10d), while bdLV.miR125b- (**Figure 4D**) and bdLV.miR93-transduced NSCs (**Figure 4E**) showed a significant and specific modulation of GFP expression during differentiation. The presence of 80–90% of GFP-expressing cells in bdLV.miRT125b-transduced stem/precursor cells and progenitors might appear counterintuitive when considering the high basal levels of miR-125b expression (see **Figure 1B**) and the 10-fold repression of GFP expression (**Figure 4B**). Given the different sensitivity of direct IF as compared to FACS analysis in setting a threshold for GFP signal and in quantifying small variations of GFP expression, it is possible that the proportion of GFP^+^ cells was slightly overestimated in IF analysis.

**Figure 5:**
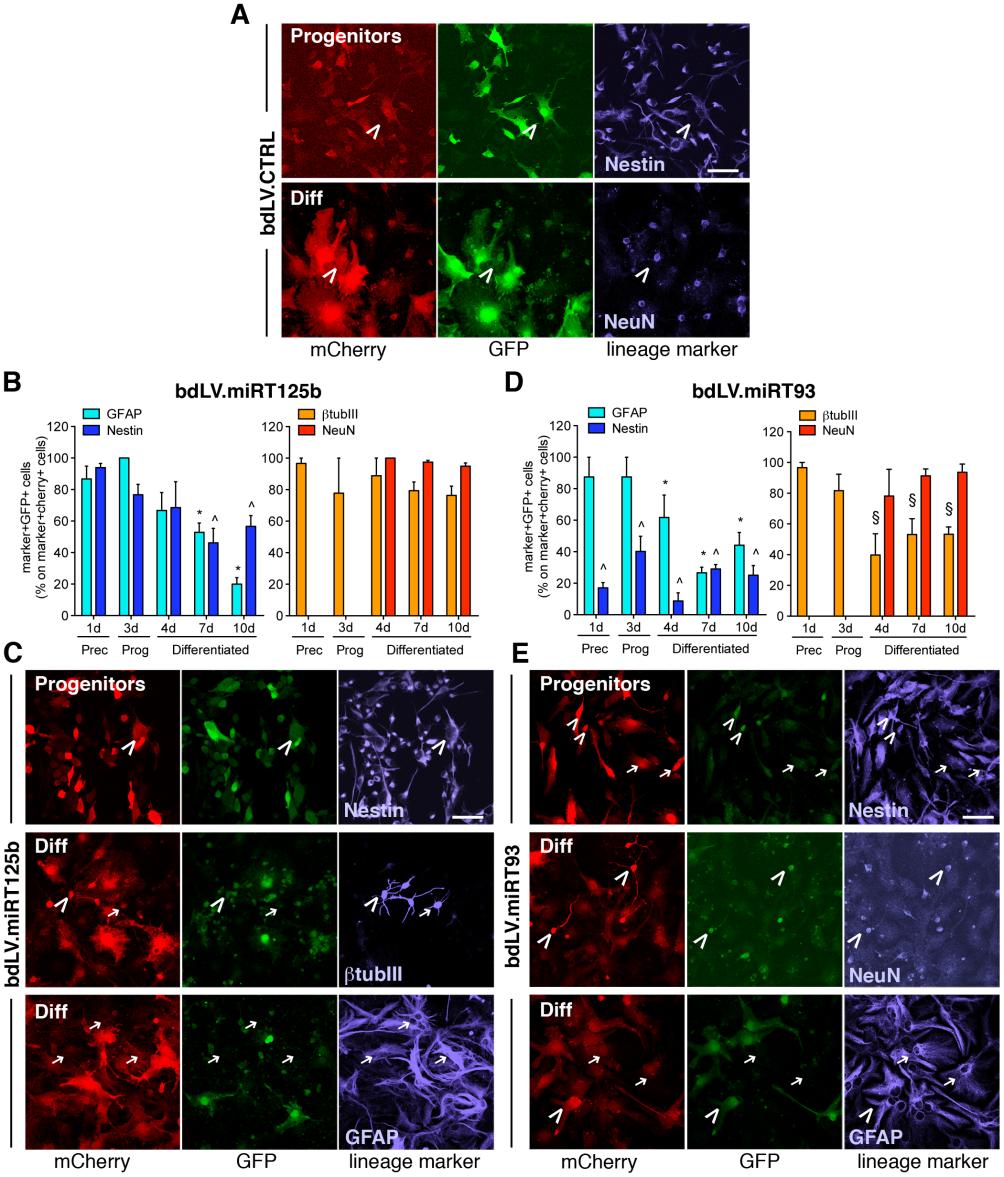
Lineage-specific modulation of miR-125b and miR-93 activity during NSC differentiation. (**A**) Representative confocal images of bdLV.CTRL-transduced NSC cultures (mCherry^+^; red, anti-mCherry antibody) showing bright GFP expression (green; direct fluorescence) in neurons (βtubIII, blue) and astroglial cells (nestin, blue). (**B,C**) Quantification and representative images of miR-125b activity in glial and neuronal subpopulations in bdLV.miRT125b-transduced NSC cultures. (**D,E**) Quantification and representative images of miR-93 activity in the glial and neuronal subpopulations in bdLV.miRT93-transduced NSC cultures. Data are the mean ±SEM; n = 2 experiments, 2–5 coverlips/antigen/experiment. Prec, *stem/precursors*; Prog, *progenitors*; Diff, *differentiated cells*; d, days in vitro. Arrowheads indicate GFP^+^marker^+^ (miR^−/low^) cells; arrows indicate GFP^−^marker^+^ (miR^+/high^) cells. Scale bars: 50 µm. Data were analyzed by one-way analysis of variance followed by Bonferroni’s posttest. *, ∧, § p<0.01 versus bdLV.CTRL-transduced GFAP^+^ cells, nestin^+^ and βtubIII^+^ cells, respectively.

We next investigated the modulation of endogenous miR-93 and miR-125b in NSC-derived cellular subpopulations. In bdLV.CTRL-transduced cultures, NSCs and their differentiated progeny expressed bright GFP (97.69±1.31% mCherry^+^GFP^+^ cells; mean ± SEM; n = 31 coverslips, 6 independent experiments) (**Figure 5A**). In bdLV.miRT125b-transduced *stem/precursors* and *progenitors*, more than 80% of nestin^+^ cells (which represent >80% of the total cell population; see **Figure S1**), co-express GFP and mCherry. Detectable GFP signal was still observed in ≈50% of nestin^+^ cells in differentiated cultures, which likely represent the persistent subpopulation of immature astroglial cells previously described in murine NSC-derived cultures [27]. Interestingly, GFP expression was low/absent in the majority of differentiated GFAP^+^ astrocytes, which represent 50–70% of the total cell population after 1 week in differentiating conditions. Altogether these results indicate upregulation of miR-125b at the time of lineage commitment and further increase of its activity as astroglial maturation progressed. Activity of miR-125b was low in both immature and mature NSC-derived neuronal populations *in vitro* (**Figure 5B, C**).

Differently from what observed in bdLV.miRT125b-transduced cultures we found persistently high activity of miR-93 in undifferentiated *stem/precursors* and in immature astroglial cells (nestin^+^) in *differentiated cultures* (**Figure 5D, E**). Expression of GFP in ∼50% of βtubIII^+^ and ∼90% of NeuN^+^ (**Figure 5D**) suggested progressive decrease of endogenous miR-93 activity in NSC-derived neurons.

The expression of the proliferation marker Ki67 was detected in 33.42±2.24% and 23.74±3.10% (% on nuclei) of *stem/precursors* and *progenitors*, respectively (mean ± SEM; n = 13), with no differences between untransduced and LV-transduced cultures (these experiments were performed using monocystronic LV.CTRL and LV.miRT; see **Figure 2A**). Expression of nestin by the vast majority (>90%) of Ki67^+^ cells indicated their immature phenotype (**Figure S2**)**.** Interestingly, GFP expression was significantly downregulated in Ki67^+^nestin^+^ cells but not in Ki67^−^nestin^+^ cells, in both LV.miRT125b- and LV.miRT93-transduced *stem/precursors* (**Figure S2**) and *progenitors* (not shown) when compared to LV.CTRL-transduced matched populations, strongly suggesting a positive association between miR-125b and miR-93 activity and proliferation of immature neural cells.

These results indicate that miR-93 and miR-125b are highly active in proliferating neural stem/precursor cells and committed progenitors, displaying a distinct time- and cell-type-pattern of modulation of both expression and activity during cell differentiation *in vitro.*

### Modulation of Endogenous miRNAs in Brain Tissues

**Figure 6:**
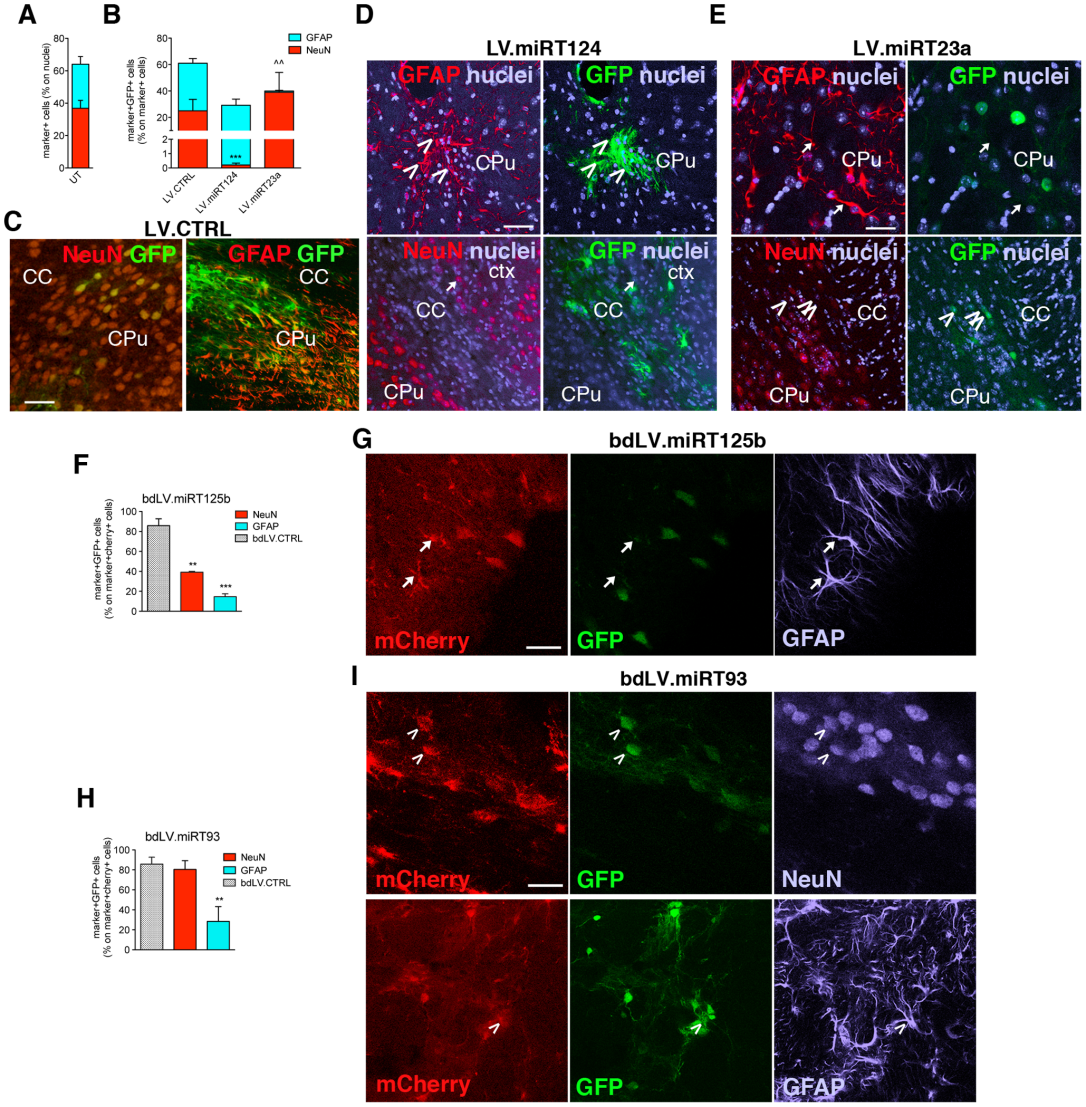
Activity of miR-124, miR-23a, miR-125b and miR-93a in striatal cell types. (**A**) Cell type composition (NeuN, neurons; GFAP, astrocytes) quantified by confocal IF analysis in striatal tissues of PND40 untransduced (UT) mice**.** (**B**) Quantitative analysis and representative confocal images of GFP expression (green, direct fluorescence) and immunoreactivity for NeuN (neurons, red) and GFAP (astrocytes, red) in brain tissue sections of PND40 mice after neonatal striatal injection of LV.CTRL (**C**), LV.miRT124 (**D**), LV.miRT23a (**E**)**.** Nuclei are counterstained with TO-PRO-3 (blue). CC, corpus callosum; CPu, Caudate Putamen**;** ctx, cortex. Scale bars: 100 µm (C–E). Data are the mean ± SEM; n = 3 animals per experimental group, 2–4 sections/animal. Data were analyzed by one-way analysis of variance followed by Bonferroni’s posttest. *** p<0.001 (NeuN), ∧∧ p<0.01 (GFAP) versus NeuN and GFAP values of LV.CTRL-injected mice. (**F, G**) Quantification and representative images of miR-125b activity after striatal injection of bdLV.miRT125b. Grey bars indicate the percentages of GFP^+^mCherry^+^ cells in bdLV.CTRL-injected mice. (**H, I**) Quantification and representative images of miR-93 activity after striatal injection of bdLV.miRT93. Arrowheads indicate GFP^+^ (miR^−/low^) cells; arrows indicate GFP^−^ (miR^+/high^) cells. Data are the mean ± SEM; n = 3 animals per experimental group, 2–4 sections/animal. Data were analyzed by one-way analysis of variance followed by Bonferroni’s posttest. **p<0.01, ***p<0.001 versus bdLV.CTRL. Scale bars: 50 µm (G, I).

In order to assess whether the modulation of miRNA activity detected *in vitro* using the sensor vector platform could be reproduced *in vivo,* we performed IF analysis on brain tissues after injection of LV.CTRL, LV.miRT124, LV.miRT23a (monocystronic LVs), bdLV.miRT125b and bdLV.miRT93 in the striatum of adult mice (**Figure 6**).

The cell type composition of the GFP^+^ cell population in LV.CTRL-injected striatal tissue closely resembled that observed in the same brain region of UT mice (**Figure 6A–C**). In the striatum of mice injected with LV.miRT124 (**Figure 6B, D**) and LV.miRT23a (**Figure 6B, E**), the expression of GFP was downregulated in >90% of NeuN^+^ neurons or GFAP^+^ astrocytes, respectively, in strict agreement with the lineage specificity of these miRNAs highlighted in NSC cultures.

Faint GFP signal characterized bdLV.miRT125b-injected striatal parenchyma, indicating high miR-125b activity in this region. Indeed, GFP expression was almost undetectable in both neurons and astrocytes (**Figure 6F, G**), pointing to high activity of this miRNA in both cell types *in vivo.* Analysis of bdLV.miRT93-injected striatal tissue indicated robust miR-93 activity in GFAP^+^ astrocytes (**Figure 6H, I**), further confirming *in vitro* data obtained for this cell population.

These data indicate good correlation between data obtained in (bd)LV.miRT-transduced NSC-derived cultures and brain tissue, in particular for miR-124 and miR-23a, whose activity is enriched in mature neurons and astrocytes, the most represented cell types in the adult striatal parenchyma.

### Modulation of miR-125b and miR-93 in the SVZ Neurogenic Niche

The cell composition and organization of the adult non-neurogenic brain tissue does not accurately reproduce the dynamic model represented by NSC compartments. The use of NSC cultures allowed us describing the modulation of miR-125b and miR-93 activity in SVZ-derived NSC populations. However, caution is necessary when extrapolating *in vitro* results to the physiological process of post-natal neurogenesis *in vivo*. Thus, we finally sought to confirm our findings by directly assessing miRNA activity in the SVZ neurogenic niche, the largest stem cell compartment in the mammalian post-natal brain [49], [50].

We injected bdLV.miRT93, bdLV.miRT125b and the monocystronic LV.CTRL into the lateral ventricles of PND2 mice. This experimental system results in efficient labelling of all the SVZ cell types, including the quiescent primary precursors (B cells; nestin^+^, GFAP^+^), the transient amplifying progenitors (C cells) and their neuronal progeny (neuroblasts, A cells; βtubIII^+^), and the SVZ astrocytes (GFAP^+^) [15]. Transgene-labelled neuroblasts will then migrate via the rostral migratory stream (RMS) to the olfactory bulb (OB), where they integrate as newly-generated post-mitotic neurons around two weeks after (bd)LV-injection.

**Figure 7:**
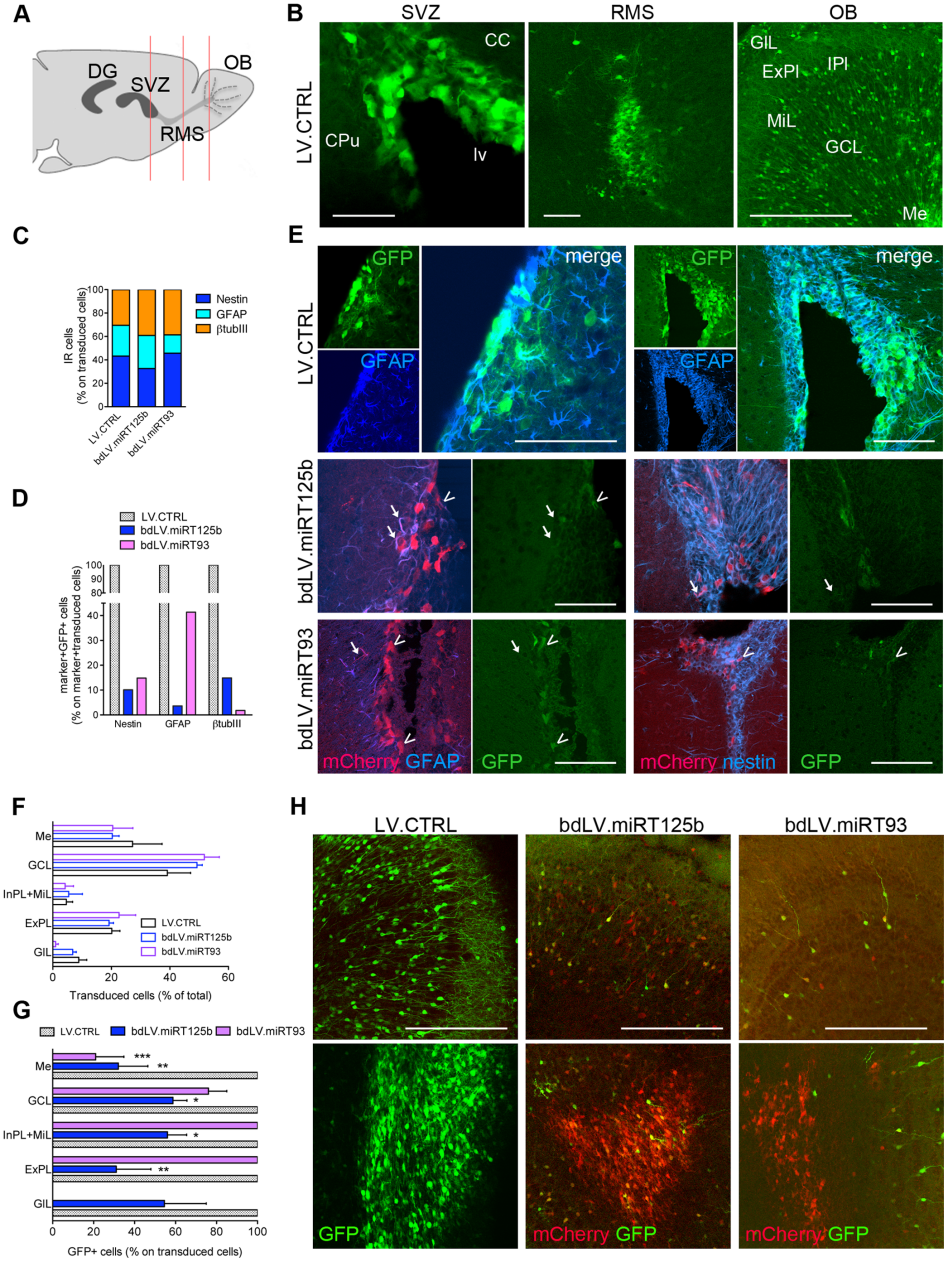
Modulation of miR-125b and miR-93 activity in the SVZ neurogenic niche. (**A**) Schematic of the neurogenic pathway in adult mice. From the SVZ stem cell niche, newly generated neuroblasts migrate along the rostral migratory stream (RMS) towards the olfactory bulb (OB), where they integrate as mature neurons. Red lines indicate the levels of the sections analyzed in this study**.** DG, dentate gyrus of the hippocampus. (**B**) Representative confocal images of PND40 mice at the level of the SVZ, RMS, OB after injection of LV.CTRL at PND2. Note the distribution of GFP^+^ neurons in the different OB layers (form dorsal to ventral: GlL, glomerular layer; ExPl, external plexiform layer; IPL, internal plexiform layer; MiL, mitral cell layer; GCL, granule cell layer; Me, medulla). CC, corpus callosum; CPu, Caudate Putamen; lv, lateral ventricle. Scale bars, 300 µm. (**C**) LV-marked cell type composition quantified at PND40 in the SVZ of LV.CTRL and bdLV.miRT-injected mice. (**D**) Downregulation of GFP expression (direct fluorescence) in the transduced (mCherry^+^) nestin^+^, GFAP^+^ or βTubIII^+^ cells in bdLV.miRT-injected mice when compared to LV.CTRL-injected mice. (**E**) Representative confocal pictures of the SVZ of PND40 mice showing robust downregulation of GFP expression in transduced (mCherry^+^) nestin^+^ and GFAP^+^ cells in LV.miRT125b and LV.miRT93-injected mice when compared to LV.CTRL-injected mice. Arrowheads indicate GFP^+^mCherry^+^marker^+^ (miR^−/low^) cells; arrows indicate GFP^−^ mCherry^+^marker^+^(miR^+/high^) cells. Scale bars, 300 µm (**F**) Distribution of transgene-labelled neurons in the different OB layers (legend as in panel B). (**G**) Downregulation of GFP expression in the transgene-labelled neuronal population of bdLV.miRT-injected mice indicates modulation of miR-125b and miR-93 activity in different OB layers. Data are the mean ± SEM. We analyzed 2–3 OB sections/mice, n = 2–4 mice/treatment group (300–3000 transduced cells). Each bdLV.miRT-treated group was compared to the LV.CTRL group by One-Way analysis of variance followed by Dunnet’s Multiple comparison test, *p<0.05, ** p<0.01, *** p<0.001 vs LV.CTRL. (**H**) Representative confocal pictures showing downregulation of GFP expression (direct fluorescence) in the transduced (mCherry^+^) neurons in the superficial (upper panel) and deeper (lower panel) OB layer in bdLV.miRT-injected mice when compared to LV.CTRL-injected mice. Scale bars, 150 µm.

We analyzed animals 40 days after injection and found transduced cells (identified by GFP and mCherry expression in LV.CTRL- and bdLV.miRT-injected mice, respectively) in the SVZ, all along the migratory pathway, and in the OB layers (**Figure 7A, B**). The relative proportions of transduced nestin^+^, GFAP^+^ and βtubIII^+^ cells in the SVZ were similar in all the treatment groups (**Figure 7C**). However, the robust downregulation of GFP expression in all SVZ cell types of bdLV.miRT125b- and bdLV.miRT93-injected mice when compared to LV.CTRL-injected mice confirmed high expression of both miRNAs in the endogenous stem/progenitor cell population from which we derived NSC-cultures (**Figure 7D, E**). We next analyzed the migration of transduced neuroblasts and their differentiation and positioning in the OB. We found a similar distribution of transgene-labelled cells in mice injected with LV.CTRL (GFP^+^ cells) and bdLV.miRT (mCherry^+^ cells) in the different OB layers (**Figure 7F**). In agreement with previous reports [51], [52], we found the majority of newly born-cells in the granule cell layer (GCL; **Figure 7F**). Of note, 60–80% of mCherry^+^ neuroblasts in the deeper OB layers (Me and GCL) strongly dowregulated GFP in bdLV.miRT93- and bdLV.miRT125b- injected mice, indicating high activity of both miRNAs in young neuroblasts during tangential migration and at the beginning of radial migration in the OB (**Figure 7G, H**). Activity of miR-93 decreased concomitantly to radial migration and differentiation of newly born neurons in the more external OB layers (**Figure 7G, H**), according to the low activity of this miRNA found in mature neurons (see **Figure 5** and **Figure 6**). On the contrary, miR-125b activity was maintained in a variable but significant proportion (30–70%) of neurons in all the OB layers (**Figure 7G, H**), similarly to what observed in bdLV.miRT125b-transduced striatal neurons (see F**igure 6**).

## Discussion

Several miRNAs have roles in NSC proliferation or in specific stages of either neuronal or glial differentiation/maturation [53]. However, the functional significance of many others remains to be elucidated. In this study we demonstrated the sensitivity and specificity of (bd)LV sensor vectors in reporting the activity of endogenous miRNAs at single cell resolution, both *in vitro* and in the complex tissue architecture of the CNS. Using this platform we described for the first time a cell type- and differentiation stage-specific modulation of miR-93 and miR-125b in NSC cultures and in the SVZ neurogenic niche, suggesting a role of these miRNAs in regulating NSC function.

By using (bd)LV sensor vectors we validated and extended previous results on the neuronal-specific miR-124 and provided new data on the activity of the astroglial-specific miR-23a. Our analysis on bdLV.miRT124-transduced NSC cultures confirmed previous data obtained using a transgenic miR-124 reporter mouse [15], demonstrating at the single cell level that SVZ-derived primary precursors lack detectable miR-124 activity, which is then upregulated in concomitance with neuronal commitment and in mature neurons. The incomplete down-regulation of GFP signal observed in the Map2^+^ cell population is likely explained by incomplete neuronal maturation in NSC-derived cultures. Similarly, the presence of heterogeneous glial cell populations in NSC-derived progeny might explain the variable pattern of miR-23a activity. In support of this hypothesis, the lineage-specific segregation of miR-124 and miR-23a activity was clearly highlighted *in vivo*, where >90% of striatal neurons and >95% of parenchymal astrocytes downregulated GFP following direct injection of LV.miRT124 and LV.miRT23a, respectively, confirming that the incorporation of target sequences for these miRNAs effectively abolished transgene expression in neurons and glial cells post-transcriptionally [22].

Genome-wide miRNA profiling on NSC cultures allowed us to shortlist miR-125b and miR-93 as candidates of potential interest in NSC biology. These miRNAs have been implicated in the control of apoptosis, proliferation and differentiation, in physiological conditions and in cancer [54], but their possible role in NSC function is poorly described. We found high basal expression levels of both miRNAs in *stem/precursors* and a good correlation between expression levels and activity during NSC differentiation in culture as well as during physiological neurogenesis *in vivo*. Importantly, the sensor approach establishes that baseline levels of these two miRNAs in NSCs are biologically meaningful and provide a high level of regulatory capacity, a conclusion which cannot be drawn from relative differences in miRNA expression levels detected by high throughput profiling techniques. That is because miRNA concentration must reach a threshold level in order become active, and this threshold is miRNA specific and not readily predictable [21], [45], [46].

The pattern of expression and activity of miR-125b in NSC cultures and *in vivo* suggested that this miRNA might regulate the transition between stem cells and committed progenitors. In agreement with reports in which upregulation of miR-125b has been associated with astrogliosis and glial cell proliferation in culture [55], we found upregulation of miR-125b activity in the astroglial compartment and in proliferating nestin^+^ cells. Interestingly, miR-125b activity in the SVZ niche was present not only in cells expressing nestin and GFAP, markers that identify primary progenitors [56], but also in βtubIII^+^ neuroblasts, which are actively proliferating in this region. We exploited neonatal intraventricular injection of bdLV.miRT to label the whole SVZ neurogenic niche, showing that miR-125b is active in a consistent fraction of newly-generated neurons in the different OB layers. Our data do not allow us to functionally distinguish the subpopulations of neurons based on miR-125b activity. SVZ neurogenesis is a continuous, asynchronous process producing thousands of new neurons per day. Thus, the downregulation of miR-125b activity might be dependent on the position of neuroblasts along the pathway and/or on the exit from the cell cycle. A more detailed study using complementary systems, i.e. transgenic reporter mice [15], could help addressing this issue.

The miR-17–92 cluster and the paralogous miR-106b-25 are emerging as key modulators of TGFβ signaling in multiple tumor types [57], [58]. Genetic ablation of these miRNAs reveals their physiologic role in the control of liver and CNS apoptosis [41], suggesting that oncogenic miRNAs could have physiological functions in somatic (stem) cells. Also, these clusters are involved in the regulation of proliferation and cell-fate decision of neural precursors in the developing neocortex [59], of self-renewal and proliferation of embryonic stem (ES) cells [60], [61], and in somatic cell reprogramming [62], [63]. Our results indicate that the basal expression of miR-93 in *stem/precursors* and *progenitors* is lower when compared to miR-125b, but its activity is up to 4-fold higher, as assessed by the high fold-repression of GFP expression in bdLV.miRT93-transduced cells. This might be related to the capacity of the other miRNAs in the cluster (which have similar seed sequences) to bind the miR-93 target sequence, thus resulting in an additive/synergic effect on miRNA activity [21], [46] **.** Both miR-93 expression and activity decrease during lineage specification and differentiation, and our expression data indicate that all the miRNAs in the cluster are modulated similarly upon NSC differentiation. These results are in agreement with a previous study [64] but seem inconsistent with an earlier study [42]. This apparent discrepancy is possibly due to the different method used to isolate and culture neural stem/progenitor cells as well as to the shorter differentiation protocol used by these authors when compared to our study.

The high miR-93 activity that we observed in GFAP^+^ cells in the SVZ niche, in NSC-cultures and in striatal tissue suggests a prominent role of miR-93 in glial cells, which include primary precursors (stem cells) and non-neurogenic parenchymal astrocytes. On the other hand, the high activity of miR-93 in SVZ neuroblasts and in Ki67^+^nestin^+^ cells in NSC cultures strongly points to the association between miR-93 and the proliferative state (as previously suggested for miR-25) [42], possibly linked to the capacity of immature progenitors to reactivate proliferation and acquire stem-like functional attributes. This ability has been described in cultured SVZ-derived transit amplifying progenitors [65], and it is a distinctive feature of astrocytes during brain development, in adult neurogenic niches, during reactive neurogenesis after brain injury or disease and also during brain tumorigenesis [66], [67], [68]. Importantly, all our data indicate a strong downregulation of miR-93 activity in mature neurons, thus underlying an important difference with respect to miR-125b.

### Conclusions

The (bd)LV.miRT platform is complementary to genome-wide PCR-based techniques that are limited to measure expression levels and that we used to shortlist miRNA candidates modulated during NSC differentiation. In this way, we identified miR-125b and miR-93 as abundantly expressed in SVZ neural stem/progenitor cells, and extended our understanding on their potential involvement in the regulation of NSC function. Gain- and loss-of-function studies combined with accurate experimental determination of true miRNA targets will clarify the role of these miRNAs in neural stem/progenitor cell biology. Exploiting the endogenous miRNA machinery can provide an alternative or a complementary strategy to de-target the expression of vector-coded transgenes in specific cell types, as recently demonstrated in *ex vivo*-hematopoietic stem cell gene therapy approach for a lysosomal storage disorder [26]. In this perspective, our work lays the framework to regulate transgene expression within the CNS, e.g. for specifically targeting the stem/progenitor cell population residing in the neurogenic niches or the differentiated cell types that are selectively affected in several neurodegenerative diseases.

## Supporting Information

Figure S1 **BdLV-transduced NSCs maintain self-renewal ability and multipotency.** (**A**) Cartoon summarizing the NSC culture system and the differentiation protocol. **(B)** Representative images showing neuronal (Map2, red) and glial progeny (GFAP, red) in NSC-derived populations during differentiation. Nuclei counterstained with DAPI (blue). Scale bar, 100 µm. **(C)** Cell counts performed after immunofluorescence analysis using lineage-specific markers showed similar cell type composition of untransduced (UT) and bdLV-transduced NSCs (bdLVs) at different stages of lineage commitment and differentiation. Data are mean ±SEM, n = 5 independent experiment, 3 independent NSC cultures, 2–4 coverlips/experiment/antigen (data from bdLV.CTRL- and bdLV.miRT-transduced cells were pooled). **(D)** Clonogenic efficiency and **(E)** long-term proliferation ability of NSCs are not altered following transduction with bdLVs. Data in (D) are the mean ± SEM, n = 5 independent experiments (data from bdLV.CTRL- and bdLV.miRT-transduced cells were pooled) Data in (E) are the mean ± SEM, n = 3 NSC independent cultures (data from bdLV.CTRL- and bdLV.miRT-transduced cells were pooled). NSCs were analyzed starting from 6 passages after transduction (total subculturing passages between 12 and 16).
(TIF)

Figure S2 **Activity of miR-125b and miR-93 in proliferating precursors and progenitors.** (A) Integrated LV genome (vector copy number, VCN) measured by qPCR in LV.CTRL-, LV.miRT125b- and LV.miRT93-transduced *stem/precursors.* The percentage of GFP^+^ cells (assessed by indirect IF analysis) was 80.53±1.1 (mean ± SEM; n = 4) in LV.CTRL-transduced cells (index of transduction efficiency). LV.miRT-transduced cells show VCN that are comparable or higher than LV.CTRL-transduced cells, suggesting comparable or even higher transduction efficiency. Data are expressed as mean ± SEM, n = 2 independent NSC lines. **(B)** Quantitative analysis of GFP expression in Ki67^+^nestin^+^ cells (on total Ki67^+^) and Ki67^−^nestin^+^ cells (on total nestin^+^) in LV.CTRL-, LV.miRT125b- and LV.miRT93-transduced *stem/precursors*. GFP^−^ cells in LV.CTRL-transduced cultures represent untranduced cells. The GFP^−^ cell population in LV.miRT-transduced *stem/precursors* is composed by a small percentage of untransduced cells while in the remaining cells GFP expression is low/absent due to the high activity of the endogenous miRNA. The proportion of GFP^+^ cells is significantly decreased in the nestin^+^Ki67^+^ cell population but not in the nestin^+^Ki67^−^ cell population as compared to LV.CTRL-transduced cells, revealing high activity of miR-125b and miR-93 in cycling precursors. Data are the mean ± SEM; n = 2 experiments, 2 NSC lines/experiment. Data were analyzed by one-way analysis of variance followed by Bonferroni’s posttest. *p<0.01 versus LV.CTRL-transduced cells. (**C**) Representative images of LV.CTRL-, LV.miRT125b- and LV.miRT93-transduced *stem/precursors* showing GFP expression in Ki67^+^Nestin^+^ cells (arrows). Arrowheads identify Ki67^+^Nestin^+^GFP^−^ cells. Scale bars, 100 µm.
(TIF)

Table S1 **miRNA expression profile in NSCs and differentiated progeny.** In order to identify novel miRNA candidates enriched and/or highly modulated in NSC-derived populations along the differentiation stages, we performed a high-throughput miRNA RT-qPCR in a time course differentiation analysis considering *stem/precursors*, *committed progenitors* and *differentiated cells* at two different stages (7d and 10d in vitro; see Figure S1). A total of 535 mammalian miRNAs were interrogated. Among them, 201 displayed detectable expression level (Ct ≤32). We used the mean expression value in a given sample to normalize high-throughput miRNA RT-qPCR data [30], [58]. Levels of miRNA expression are expressed as ΔCt.
(PDF)

Table S2 **Heatmap of the most variable top-ranked miRNAs.** Heatmap showing the list of miRNAs that are modulated along the differentiation process. Data are expressed as ΔCt normalized on mean expression value. We assigned an arbitrary color code referring to the relative abundance of each miRNA. We reported miRNAs that displayed differential expression (ΔΔCt ≥1) in *progenitors* and/or *differentiated cells* as compared to *stem/precursors*. #1 and #2 indicate two independent NSC lines.
(PDF)

Methods S1 (DOCX)
