# Supplementary figures and images for: Dynamic Activity of miR-125b and miR-93 during Murine Neural Stem Cell Differentiation In Vitro and in the Subventricular Zone Neurogenic Niche
Source: PLoS One. 2013 Jun 27;8(6):e67411. doi: 10.1371/journal.pone.0067411 (PMC3694868; doi:10.1371/journal.pone.0067411)

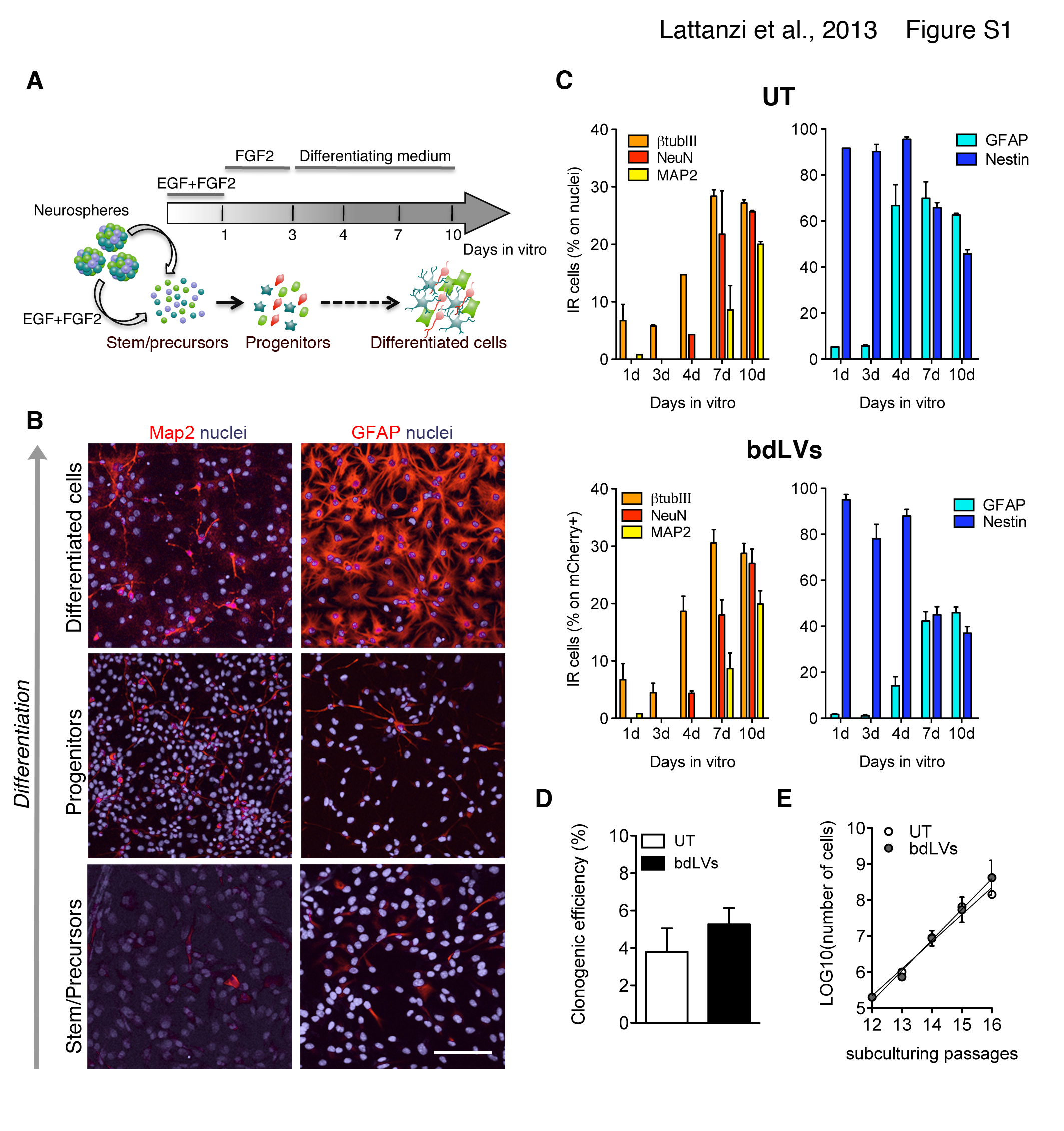

Supplement: Figure S1 — BdLV-transduced NSCs maintain self-renewal ability and multipotency. (A) Cartoon summarizing the NSC culture system and the differentiation protocol. (B) Representative images showing neuronal (Map2, red) and glial progeny (GFAP, red) in NSC-derived populations during differentiation. Nuclei counterstained with DAPI (blue). Scale bar, 100 µm. (C) Cell counts performed after immunofluorescence analysis using lineage-specific markers showed similar cell type composition of untransduced (UT) and bdLV-transduced NSCs (bdLVs) at different stages of lineage commitment and differentiation. Data are mean ±SEM, n = 5 independent experiment, 3 independent NSC cultures, 2–4 coverlips/experiment/antigen (data from bdLV.CTRL- and bdLV.miRT-transduced cells were pooled). (D) Clonogenic efficiency and (E) long-term proliferation ability of NSCs are not altered following transduction with bdLVs. Data in (D) are the mean ± SEM, n = 5 independent experiments (data from bdLV.CTRL- and bdLV.miRT-transduced cells were pooled) Data in (E) are the mean ± SEM, n = 3 NSC independent cultures (data from bdLV.CTRL- and bdLV.miRT-transduced cells were pooled). NSCs were analyzed starting from 6 passages after transduction (total subculturing passages between 12 and 16). (TIF) [file pone.0067411.s001.tif]

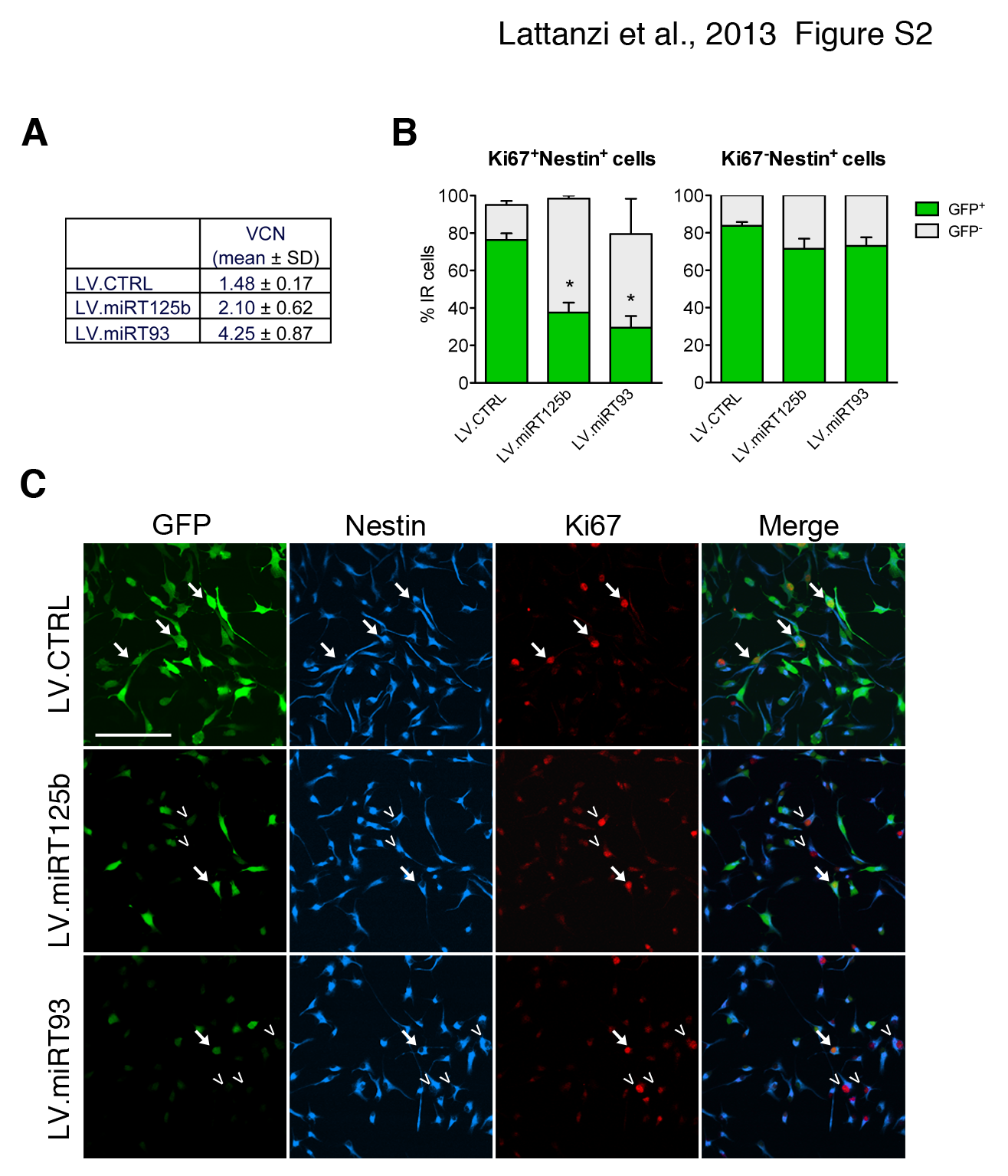

Supplement: Figure S2 — Activity of miR-125b and miR-93 in proliferating precursors and progenitors. (A) Integrated LV genome (vector copy number, VCN) measured by qPCR in LV.CTRL-, LV.miRT125b- and LV.miRT93-transduced stem/precursors. The percentage of GFP+ cells (assessed by indirect IF analysis) was 80.53±1.1 (mean ± SEM; n = 4) in LV.CTRL-transduced cells (index of transduction efficiency). LV.miRT-transduced cells show VCN that are comparable or higher than LV.CTRL-transduced cells, suggesting comparable or even higher transduction efficiency. Data are expressed as mean ± SEM, n = 2 independent NSC lines. (B) Quantitative analysis of GFP expression in Ki67+nestin+ cells (on total Ki67+) and Ki67−nestin+ cells (on total nestin+) in LV.CTRL-, LV.miRT125b- and LV.miRT93-transduced stem/precursors. GFP− cells in LV.CTRL-transduced cultures represent untranduced cells. The GFP− cell population in LV.miRT-transduced stem/precursors is composed by a small percentage of untransduced cells while in the remaining cells GFP expression is low/absent due to the high activity of the endogenous miRNA. The proportion of GFP+ cells is significantly decreased in the nestin+Ki67+ cell population but not in the nestin+Ki67− cell population as compared to LV.CTRL-transduced cells, revealing high activity of miR-125b and miR-93 in cycling precursors. Data are the mean ± SEM; n = 2 experiments, 2 NSC lines/experiment. Data were analyzed by one-way analysis of variance followed by Bonferroni’s posttest. *p<0.01 versus LV.CTRL-transduced cells. (C) Representative images of LV.CTRL-, LV.miRT125b- and LV.miRT93-transduced stem/precursors showing GFP expression in Ki67+Nestin+ cells (arrows). Arrowheads identify Ki67+Nestin+GFP− cells. Scale bars, 100 µm. (TIF) [file pone.0067411.s002.tif]
