# Supplementary material for: Dynamic Activity of miR-125b and miR-93 during Murine Neural Stem Cell Differentiation In Vitro and in the Subventricular Zone Neurogenic Niche
Source: PLoS One. 2013 Jun 27;8(6):e67411. doi: 10.1371/journal.pone.0067411 (PMC3694868; doi:10.1371/journal.pone.0067411)
Supplement: Table S1 — miRNA expression profile in NSCs and differentiated progeny. In order to identify novel miRNA candidates enriched and/or highly modulated in NSC-derived populations along the differentiation stages, we performed a high-throughput miRNA RT-qPCR in a time course differentiation analysis considering stem/precursors, committed progenitors and differentiated cells at two different stages (7d and 10d in vitro; see Figure S1). A total of 535 mammalian miRNAs were interrogated. Among them, 201 displayed detectable expression level (Ct ≤32). We used the mean expression value in a given sample to normalize high-throughput miRNA RT-qPCR data [30], [58]. Levels of miRNA expression are expressed as ΔCt. (PDF) [file pone.0067411.s003.pdf]

Supplementary Table 1. MiRNA expression profile in NSCs and differentiated progeny.

	STEM/PRECURSORS (1d)		PROGENITORS (3d)		DIFFERENTIATED CELLS (7d)		DIFFERENTIATED CELLS (10d)	
let-7a	3.2359	2.8104	3.0076	3.1209	2.9552	3.2489	3.2248	3.2225
let-7a#	-1.1334	-1.4656	-0.8674	-0.7001	-0.3443	-1.8405	-0.0262	-0.5624
let-7b	5.7652	4.9717	5.5027	5.9206	5.9867	6.1210	5.7099	6.1397
let-7b	NA	NA	NA	NA	NA	NA	NA	NA
let-7b#	NA	NA	NA	NA	NA	NA	NA	NA
let-7c	6.5513	5.7931	6.2325	6.5369	6.7511	6.9043	6.6469	6.9816
let-7c-1#	-2.9338	-2.6659	-1.2723	-2.1617	-2.3510	-3.4820	-2.2812	-2.3530
let-7d	4.5266	4.1174	4.1629	4.2279	4.2296	3.9956	4.0599	4.3113
let-7d#	-0.8420	NA	NA	NA	NA	NA	NA	NA
let-7e	6.1171	5.5133	5.5928	5.5586	5.9220	5.9154	5.9361	6.1068
let-7f	1.5164	1.3939	1.3295	1.6230	1.5712	1.6299	1.7758	1.9351
let-7f-1#	NA	NA	NA	NA	NA	NA	NA	-5.5536
let-7g	3.4496	2.9653	3.2656	3.3312	3.3291	3.1162	3.4949	3.2948
let-7g#	NA	NA	-3.6813	NA	NA	NA	NA	NA
let-7i	3.8633	3.1837	3.8069	3.7258	4.1623	3.7385	4.3820	3.8801
let-7i#	NA	NA	NA	NA	NA	NA	NA	NA
mir-1	NA	NA	NA	NA	NA	NA	NA	NA
mir-100	3.8239	2.8618	3.7566	3.8020	4.0689	4.2344	4.2848	4.5449
mir-101 (TM 2253)	0.6395	0.3346	0.4969	0.4813	0.4324	-0.5181	0.3284	-0.1523
mir-101a#	NA	NA	NA	NA	NA	NA	NA	NA
mir-101b	0.7753	0.4498	1.0153	0.8661	0.7015	-0.1830	0.6248	0.2220
mir-103	0.9099	0.5117	0.5404	0.6819	0.5829	0.6231	0.8764	0.5905
mir-105	NA	NA	NA	NA	NA	NA	NA	NA
mir-106a	5.9542	5.0307	5.2177	4.6508	5.2626	3.7228	5.2221	2.9982
mir-106b	3.2228	3.1798	3.3599	3.3643	2.6247	2.2685	2.6643	2.0930
mir-106b#	0.4833	-0.7821	-0.0684	-0.6407	-1.8859	NA	-1.1606	-2.2225
mir-107	-3.1589	-3.0320	-3.1271	-3.0467	-3.1192	-3.3918	-3.6888	-3.3664
mir-10a	NA	NA	NA	NA	NA	NA	NA	NA
mir-10a#	NA	NA	NA	NA	NA	NA	NA	NA
mir-10b	NA	NA	NA	NA	NA	NA	NA	NA
mir-10b#	NA	NA	NA	NA	NA	NA	NA	NA
mir-122	NA	NA	NA	NA	NA	NA	NA	NA
mir-124#	NA	NA	NA	NA	NA	NA	NA	NA
mir-124a (TM 1182)	-3.3297	NA	NA	NA	NA	NA	NA	NA
mir-124a (TM446)	NA	NA	NA	NA	NA	NA	NA	NA
mir-125a-3p	-3.0564	-3.0803	-4.9960	-4.6960	-3.6667	-4.5240	-4.8538	-4.2930
mir-125a-5p	4.2383	4.0720	3.9011	4.1164	4.3134	4.4094	4.2389	4.5689
mir-125b (TM 449)	5.8679	5.2445	6.0992	6.5100	6.4739	6.5511	6.3057	6.7888
mir-125b-1#	-3.8466	-4.1492	-4.3349	-3.8414	-3.8567	-4.3703	9.3749	-3.8040
mir-125b#	-4.0195	-4.8968	-4.0974	-3.9799	-3.3680	-4.0155	-3.0371	-2.1639
mir-126 (TM 2228)	-2.2230	-2.9090	-3.1384	-3.0484	-3.4467	-3.5954	-3.7029	-3.8617
mir-126#	-4.5689	-4.3284	-4.7109	NA	-4.7123	NA	NA	NA
mir-127	-2.3291	NA	-4.0574	NA	-3.2364	NA	-3.7892	NA
mir-127-5p	NA	NA	NA	NA	NA	NA	NA	NA
mir-128a	1.3267	0.5634	0.8825	0.4981	-0.0071	-0.5276	0.0223	-0.8040
mir-129 (TM 590)	-1.8477	-1.7255	-3.7235	-4.5856	-4.1577	-4.6716	NA	-4.2146
mir-129-3p	2.0724	2.3126	1.0346	0.3588	0.7225	0.4058	0.3178	0.8863
mir-130a	2.1684	1.8861	2.3639	2.6190	2.0296	1.2663	2.3579	2.1896
mir-130b	1.2122	-0.1839	0.7574	-0.5233	0.4424	-1.9588	0.2922	-1.2642
mir-130b#	1.3285	0.0889	0.7267	-0.7710	-0.1294	-2.5194	0.2074	-1.8602
mir-132	4.5919	4.4867	2.0439	2.0230	1.6494	2.6068	1.9796	2.6100
mir-133a (TM 2246)	NA	NA	NA	NA	NA	NA	NA	NA
mir-133a (TM 458)	NA	NA	NA	NA	NA	NA	NA	NA
mir-133a#	NA	NA	NA	NA	NA	NA	NA	NA
mir-133b	NA	NA	NA	NA	NA	NA	NA	NA
mir-134	NA	NA	NA	NA	NA	NA	NA	NA
mir-135a	-0.2589	-4.5107	-0.2466	-1.1238	-2.0306	-2.7412	-2.0643	-0.6131
mir-135b	-2.7991	-4.7926	-2.7907	-3.4912	-3.3060	-3.9807	-3.7251	-3.0403
mir-136	NA	NA	NA	NA	NA	NA	NA	NA
mir-136#	NA	NA	NA	NA	NA	NA	NA	NA
mir-137	-4.0869	NA	-4.4716	NA	-5.0342	NA	-4.2625	-5.4096
mir-138	1.0310	-0.4006	1.3869	0.0380	5.6017	0.4491	6.0122	2.1820
mir-138#	-4.6956	NA	NA	NA	0.4832	-5.2314	0.7624	-3.8279
mir-139-3p	NA	NA	NA	NA	NA	NA	NA	NA
mir-139-5p	4.0846	3.3789	3.0970	2.4774	0.7410	1.1075	-0.3199	0.4343
mir-140	0.7262	0.1354	0.7939	0.9270	1.8793	2.7391	1.8910	3.2769
mir-140-3p (TM 223	-3.8994	-4.3888	-3.8075	-4.2861	-3.4093	-2.2995	NA	-2.0740
mir-141	NA	NA	NA	NA	NA	NA	NA	NA
mir-141#	-2.4745	-1.8221	-1.9210	-2.2219	-1.6076	-3.6639	-1.9122	-2.0074
mir-142-3p	NA	NA	NA	NA	NA	NA	NA	NA
mir-142-5p	NA	NA	NA	NA	NA	NA	NA	NA
mir-143	NA	NA	NA	-4.8111	-4.9537	-4.2441	NA	NA
mir-145	-4.7815	-3.7689	-3.7878	-1.6375	-1.5688	-0.4314	-1.1049	-0.3071
mir-145#	NA	NA	NA	NA	NA	NA	NA	NA
mir-146a	0.5663	2.0656	1.1398	2.4811	-0.1531	1.2592	-0.5045	-1.4879
mir-146b	0.5144	0.6122	0.6260	0.6585	1.5248	1.7948	1.6517	1.3936
mir-146b#	NA	NA	NA	NA	NA	NA	NA	NA
mir-147b (TM 2262)	NA	NA	NA	NA	NA	NA	NA	NA
mir-148a	-2.4831	-2.3421	-2.2161	-1.7409	-2.2544	-2.6655	-2.8582	-3.0577
mir-148a#	NA	NA	NA	NA	NA	NA	NA	NA
mir-148b	-3.6243	-3.8494	-3.0124	-3.3708	-2.8102	-3.0280	-2.4846	-2.7242
mir-149	4.7003	3.8778	4.3724	4.4167	4.2204	4.6509	4.5225	4.8268
mir-150	NA	-4.8775	NA	-4.8495	NA	NA	NA	NA

mir-150#	NA	NA	NA	NA	NA	NA	NA	NA
mir-151	-0.1856	-0.8570	-0.6450	-1.0538	-0.7236	-1.6218	-0.8627	-1.3493
mir-152	-1.9715	-1.9418	-1.3819	-0.4542	0.8235	0.9870	1.0252	1.7790
mir-153	NA	NA	NA	NA	NA	NA	NA	NA
mir-154	NA	NA	NA	NA	NA	NA	NA	NA
mir-154#	NA	NA	NA	NA	NA	NA	NA	NA
mir-155	0.3293	0.5975	0.3738	-0.5682	0.2987	0.5212	0.1224	0.6220
mir-15a	-0.5097	-1.1229	-0.6317	-0.6287	-0.6035	-0.7062	-0.5699	-0.8159
mir-15a#	-1.1376	-2.2427	-2.0321	-2.3058	-2.3219	-3.5060	-2.1122	-2.2803
mir-15b	3.9392	3.8662	3.3500	2.9645	2.4651	2.2538	2.5421	2.3562
mir-15b#	-0.5501	-0.2087	-1.3026	NA	NA	NA	NA	NA
mir-16	6.5949	5.9544	6.0170	5.8200	5.3033	5.5394	5.2740	5.4335
mir-16#	-4.9660	NA	-4.3512	-5.5674	-4.5565	NA	-4.7697	NA
mir-17	5.7221	5.0175	5.0480	4.8328	5.2164	3.5299	5.0236	2.9466
mir-17#	NA	NA	NA	NA	NA	NA	-4.1638	NA
mir-181a	2.3191	2.0241	2.5348	2.7337	2.2453	2.5327	2.8617	1.7591
mir-181c	-2.6553	-3.0544	-2.1144	-2.0101	-2.5230	-2.8917	-2.6075	-2.9771
mir-182	NA	-4.5947	NA	NA	NA	-5.5560	NA	NA
mir-183	NA	NA	NA	NA	NA	NA	NA	NA
mir-183#	NA	NA	NA	NA	NA	NA	NA	NA
mir-184	-2.2689	-2.5412	-2.9966	-3.2765	NA	-3.8977	-4.9664	-4.7314
mir-185	-2.4296	-3.2009	-2.4959	-2.2005	-0.7890	-1.1491	-0.9288	-0.4461
mir-186	0.6937	-0.3174	0.2388	0.2134	0.2914	-0.3368	0.3517	0.2052
mir-186#	-2.6231	-2.7982	-2.7954	-2.8433	-2.9015	-4.2290	-2.6616	-3.8415
mir-187	-2.1009	NA	-2.7086	-2.7380	-1.7184	-1.3972	NA	-2.4542
mir-188-3p	NA	NA	NA	NA	NA	NA	NA	NA
mir-188-5p	-1.3013	-0.3842	-1.5679	-1.4199	-1.3993	0.5867	-0.9428	0.1268
mir-18a	0.6284	0.4713	0.3942	-0.1683	0.1698	-2.1196	-0.1255	-2.7675
mir-18a#	-2.4421	-3.0193	-3.6797	-4.5197	-3.0912	NA	-3.0223	NA
mir-18b	NA	NA	NA	NA	NA	NA	NA	NA
mir-190	NA	-4.5666	-5.0044	-4.1737	-4.0006	-3.8004	-3.9058	-3.6596
mir-190b	-2.4320	-2.6007	-2.9096	-2.5532	-1.9293	-3.3121	-2.1375	-1.2264
mir-191	3.9841	4.3737	4.0865	4.8166	4.1049	4.4769	4.0912	4.6224
mir-191#	NA	-4.8417	NA	-5.3002	-5.1453	NA	-5.0519	-5.0946
mir-192	-1.8050	-2.9391	-1.7351	-2.7422	-1.1826	-1.2789	-1.1552	-1.4268
mir-193#	-0.4533	-0.1864	-2.7886	-3.4626	-2.1212	-2.8153	-2.0176	-1.9733
mir-193a-3p (TM 22	NA	NA	NA	NA	NA	NA	NA	NA
mir-193b	2.9348	2.6144	2.7052	2.5448	3.5196	3.1721	3.9648	4.4469
mir-194	-3.5071	-4.5804	-3.2081	-3.1875	-2.2426	-2.9292	-1.8836	-2.4366
mir-195	2.2740	1.3904	1.9180	1.8822	1.7968	1.7190	1.7832	2.4260
mir-196a#	NA	NA	NA	NA	NA	NA	NA	NA
mir-196b	NA	NA	NA	NA	NA	NA	NA	NA
mir-197	NA	NA	NA	NA	-4.6305	NA	NA	NA
mir-199a (TM 498)	NA	NA	NA	NA	NA	NA	0.6546	NA
mir-199a-3p	-4.1649	-5.2029	-3.8158	NA	-3.6266	NA	-4.3323	-4.8507
mir-199b	NA	NA	NA	NA	NA	NA	NA	NA
mir-19a	0.8194	0.6369	0.4377	0.1164	0.8982	-1.5081	0.4648	-1.2844
mir-19a#	NA	NA	NA	NA	NA	NA	NA	NA
mir-19b	5.5164	5.1783	5.4218	5.1192	5.5901	3.7608	5.4028	3.7008
mir-200a	NA	NA	NA	NA	NA	NA	NA	NA
mir-200a#	NA	NA	NA	NA	NA	NA	NA	NA
mir-200b	NA	NA	NA	NA	NA	NA	NA	NA
mir-200b#	NA	NA	NA	NA	NA	NA	NA	NA
mir-200c	NA	NA	NA	NA	NA	NA	NA	NA
mir-200c#	NA	NA	NA	NA	NA	NA	NA	NA
mir-201	NA	NA	NA	NA	NA	NA	NA	NA
mir-202	-4.7274	NA	-4.4544	NA	-3.9027	-3.7299	NA	-3.9714
mir-202-5p	NA	NA	NA	NA	NA	NA	NA	NA
mir-203#	NA	NA	NA	NA	NA	NA	NA	NA
mir-204	-1.0505	-3.5959	-1.0624	-1.8374	-2.8281	-3.8145	-3.8583	-3.6728
mir-205	NA	NA	NA	NA	NA	NA	NA	NA
mir-206	-2.9780	-4.7546	-2.3267	-3.6099	-2.9906	-4.0115	-1.9686	-5.0299
mir-207	NA	NA	NA	NA	NA	NA	NA	NA
mir-208	NA	NA	NA	NA	NA	NA	NA	NA
mir-208b	NA	NA	NA	NA	NA	NA	NA	NA
mir-20a	5.6752	5.0792	5.4283	4.8079	5.0813	3.4348	5.1511	2.7106
mir-20a#	-2.4829	NA	NA	NA	NA	NA	NA	NA
mir-20b	2.7924	2.1254	2.2833	1.4586	2.3088	0.5182	1.8879	-0.4939
mir-20b#	NA	NA	NA	NA	NA	NA	NA	NA
mir-21	2.7589	3.3613	3.7824	3.8824	3.4032	3.8482	3.4759	4.7155
mir-21#	-3.2145	-3.5435	NA	NA	-4.8787	-4.1526	NA	-2.7920
mir-210	0.6551	-2.3466	-1.5627	NA	NA	NA	NA	-1.7386
mir-211	NA	NA	NA	NA	NA	NA	NA	NA
mir-212	2.5065	2.6914	-0.0823	0.0262	-0.3568	0.8850	0.3283	0.7710
mir-214	NA	NA	NA	NA	NA	NA	NA	NA
mir-214 (TM 2306)	NA	NA	NA	NA	NA	NA	-0.0655	NA
mir-214#	NA	NA	NA	NA	NA	NA	NA	NA
mir-215	NA	NA	NA	NA	NA	NA	NA	NA
mir-216a	NA	NA	NA	NA	NA	NA	NA	NA
mir-216b	NA	NA	NA	NA	NA	NA	NA	NA
mir-217	NA	NA	NA	NA	NA	NA	NA	NA
mir-218	-1.2252	-5.0842	-1.9248	-4.3755	-0.4875	NA	0.5798	-5.2917
mir-218-1#	NA	NA	NA	NA	NA	NA	NA	NA
mir-218-2#	NA	NA	NA	NA	NA	NA	NA	NA

mir-219	NA	NA	NA	NA	-2.4020	NA	-2.1853	NA
mir-219	NA	NA	NA	NA	-1.8791	NA	-1.8362	NA
mir-22	-1.3177	0.2872	0.6973	0.7001	1.6960	1.2680	2.2001	2.6925
mir-22#	-1.6240	-1.4593	-1.3518	-1.8613	-0.1927	-1.6004	0.0027	-0.1228
mir-220	NA	NA	NA	NA	NA	NA	NA	NA
mir-221	2.3582	2.9574	2.3396	2.0442	3.4978	3.5778	3.5115	3.9182
mir-222	3.8746	4.2444	3.4775	3.2974	4.2642	4.4191	4.1499	4.2819
mir-224	NA	NA	NA	NA	NA	NA	NA	NA
mir-23a	NA	NA	NA	NA	NA	NA	NA	NA
mir-23b	-3.1519	-4.5549	-4.2091	-2.6480	-1.4240	-1.9141	-1.1189	-2.0665
mir-24	4.0707	4.9343	3.9406	4.4770	5.5411	6.4997	5.8348	6.7214
mir-24-2#	-2.3099	-1.1344	-2.5156	-2.1215	-0.5985	-0.2522	-0.3590	0.6830
mir-25	1.9660	1.0947	1.0659	0.6615	0.3860	0.5884	0.8406	0.4953
mir-26a	4.8693	3.6465	4.5973	4.9720	5.3748	5.0886	5.0819	5.5567
mir-26b	1.8851	2.0393	2.3171	2.8042	2.6228	3.0940	2.7483	3.3999
mir-26b#	-3.6506	-3.9559	-3.8192	-3.8862	-3.2753	-3.9808	-3.2418	-3.2898
mir-27a	-0.7330	0.6142	-0.4785	0.2825	0.7762	2.0040	1.3342	2.1494
mir-27a#	-1.7024	0.0498	-5.2357	NA	-4.8441	-3.4704	-4.1565	-3.8107
mir-27b	0.0650	0.1148	-0.0077	0.1307	1.6704	1.7433	2.2632	1.8178
mir-27b#	NA	NA	NA	NA	-3.7532	-5.4343	-3.5713	NA
mir-28	-0.0043	-0.8594	0.0731	-0.2796	-0.1606	-0.1577	-0.3836	0.1801
mir-28#	-2.7551	-3.3948	-2.5915	-2.7744	-2.7467	NA	-3.1060	-2.3567
mir-290-3p	NA	NA	NA	NA	NA	NA	NA	NA
mir-290-5p	NA	NA	NA	NA	NA	NA	NA	NA
mir-291-5p	NA	NA	NA	NA	NA	NA	NA	NA
mir-291a-3p	NA	NA	NA	NA	NA	NA	NA	NA
mir-291b-3p	NA	NA	NA	NA	NA	NA	NA	NA
mir-291b-5p	NA	NA	NA	NA	NA	NA	NA	NA
mir-292-3p	NA	NA	NA	NA	NA	NA	NA	NA
mir-292-5p	NA	NA	NA	NA	NA	NA	NA	NA
mir-293	NA	NA	NA	NA	NA	NA	NA	NA
mir-293#	NA	NA	NA	NA	NA	NA	NA	NA
mir-294	NA	NA	NA	NA	NA	NA	NA	NA
mir-294#	NA	NA	NA	NA	NA	NA	NA	NA
mir-295	NA	NA	NA	NA	NA	NA	NA	NA
mir-295#	NA	NA	NA	NA	NA	NA	NA	NA
mir-296 (TM 527)	1.5247	1.5463	1.2299	1.6453	1.4482	1.2643	1.8224	1.7768
mir-296-3p	-2.2433	NA	-3.4484	NA	NA	NA	-4.7994	NA
mir-297a#	-3.2094	-4.0766	-3.3828	-2.9966	-3.2406	-3.2521	-2.8237	-3.0440
mir-297b	NA	NA	NA	NA	NA	NA	NA	NA
mir-297c	NA	NA	NA	NA	NA	NA	NA	NA
mir-298	0.9506	-0.1377	0.3962	-0.2685	0.5108	-0.8258	0.0312	-1.0856
mir-299	NA	NA	NA	NA	NA	NA	NA	NA
mir-299-5p (TM 600	NA	NA	NA	NA	NA	NA	NA	NA
mir-29a	5.0550	5.5347	4.4071	4.0977	4.1145	4.2602	4.2510	4.7453
mir-29a#	-2.4606	-2.0447	-2.9408	-2.9919	-2.9936	-4.9162	-2.7106	-3.3427
mir-29b	-2.6383	-2.4339	-3.7850	-4.3426	-4.1428	-4.8106	-3.2339	-3.8024
mir-29b#	1.7341	1.9482	-0.9004	-1.6482	-1.1871	-2.0764	-0.8972	-1.3932
mir-29c#	NA	NA	NA	-5.5246	-4.9812	NA	-4.6802	-4.8951
mir-300	NA	NA	NA	NA	NA	NA	NA	NA
mir-300#	NA	NA	NA	NA	NA	NA	NA	NA
mir-301 (TM 528)	1.0797	0.2417	0.4205	0.7412	0.6638	-0.4779	0.6103	-0.3700
mir-301b	1.3616	0.8304	0.7413	1.0832	1.0138	-0.0497	1.0431	0.2516
mir-302a	NA	NA	NA	NA	NA	NA	NA	NA
mir-302a#	NA	NA	NA	NA	NA	NA	NA	NA
mir-302b	NA	NA	NA	NA	NA	NA	NA	NA
mir-302b#	NA	NA	NA	NA	NA	NA	NA	NA
mir-302c	NA	NA	NA	NA	NA	NA	NA	NA
mir-302c#	NA	NA	NA	NA	NA	NA	NA	NA
mir-302d	NA	NA	NA	NA	NA	NA	NA	NA
mir-30a-3p	3.2339	2.4382	2.9241	3.5666	3.6593	3.6065	3.8031	4.3019
mir-30a-5p	1.5709	1.4833	1.7133	1.8188	2.1571	2.3477	2.7281	2.8727
mir-30b#	NA	NA	NA	NA	NA	NA	NA	NA
mir-30c	5.3797	5.0195	5.3604	5.6446	5.5411	5.7403	5.8462	6.2823
mir-30c-1#	NA	NA	NA	NA	NA	NA	NA	NA
mir-30c-2#	NA	NA	NA	NA	NA	NA	NA	NA
mir-30d	-0.3238	-0.5018	-0.1835	-0.0641	0.3527	0.1633	0.6227	0.9025
mir-30e	1.0903	0.7164	1.1266	1.4976	1.5516	1.3034	1.8733	1.8364
mir-30e-3p	3.5669	3.2596	3.5255	3.8853	3.9840	3.5338	4.4472	4.6251
mir-31	1.8921	3.4223	0.5572	1.4770	1.2637	3.5937	1.3848	1.8420
mir-31#	0.7428	1.6719	-1.4835	-0.4033	-0.0657	0.1325	0.2762	0.3104
mir-32	NA	NA	NA	NA	NA	NA	NA	NA
mir-320	2.7531	1.8273	1.9802	1.8980	1.9511	1.7704	1.9461	2.3196
mir-322#	1.3359	-0.8261	0.8255	-0.3802	-0.2936	-0.5289	-0.6754	-0.8016
mir-323-3p	NA	NA	NA	NA	NA	NA	NA	NA
mir-324-3p	0.1583	-0.2968	-0.1239	-0.2437	-0.5169	-0.9841	-0.4868	-0.4658
mir-324-5p	1.0836	0.2995	0.6972	0.7441	1.0761	0.5715	1.2250	0.5538
mir-325 (TM 1060)	-2.6504	-3.7005	-3.3460	-3.6218	-2.5830	-3.8550	-3.1315	-2.5129
mir-325 (TM 2510)	-2.1434	-2.4990	-2.4533	-2.8718	-2.2076	-2.8035	NA	-2.8588
mir-326	-4.8361	NA	-4.8474	-5.0397	-3.8278	-5.3663	-3.5627	-4.6769
mir-327	NA	NA	NA	NA	NA	NA	NA	NA
mir-328	3.9291	4.0570	4.1929	4.8573	5.0360	4.7192	4.8865	5.0711
mir-329	NA	NA	NA	NA	NA	NA	NA	NA
mir-330	NA	NA	NA	NA	NA	NA	NA	NA

mir-330-5p	NA	NA	NA	NA	NA	NA	NA	NA
mir-331 (TM 545)	2.0311	1.5820	2.3059	2.2926	2.7408	2.2355	2.7404	2.4027
mir-331-5p	-3.9295	-3.8252	-4.0606	-3.4722	-4.1031	NA	-4.2380	-4.7120
mir-335 (TM 546)	3.3744	3.4564	3.6258	3.3812	2.1961	1.7786	1.7414	1.6895
mir-335#	1.6965	2.4478	1.4365	0.6534	-0.6127	-0.8245	-0.7127	-1.2033
mir-337-3p	NA	NA	NA	NA	NA	NA	NA	NA
mir-337-5p	NA	NA	NA	NA	NA	NA	NA	NA
mir-338-3p	NA	NA	NA	-4.9106	-2.6976	NA	-2.2394	NA
mir-339-3p	NA	NA	NA	NA	NA	NA	NA	NA
mir-339-5p	NA	NA	NA	-2.1458	-2.6687	NA	NA	NA
mir-33a (TM 2135)	NA	NA	NA	NA	NA	NA	NA	NA
mir-33a# (TM 2136)	-3.0522	-3.7785	-3.5101	-3.6636	-3.6402	-3.3740	-3.0255	-3.1064
mir-340 (TM 2258)	-0.2455	-0.8226	-0.0123	-0.0614	0.4080	-1.0072	0.4691	-0.6489
mir-340#	-0.4591	-1.0550	-0.9405	-0.6026	-0.1003	-0.9605	-0.0501	-0.8960
mir-342-3p	1.8231	1.0004	1.0910	1.1483	1.4391	0.4526	1.3846	0.7722
mir-342-5p	-3.8286	NA	NA	NA	NA	NA	NA	NA
mir-343	NA	NA	NA	NA	NA	NA	NA	NA
mir-344	-0.1642	-0.4111	0.2083	0.4212	0.3364	0.3341	0.5975	1.0393
mir-345-3p	-4.9858	NA	NA	NA	NA	NA	NA	NA
mir-345-5p	-3.1533	-4.5594	-3.2264	-3.4718	-2.6680	-3.5214	-2.9622	-2.5935
mir-346	NA	NA	NA	NA	NA	NA	NA	NA
mir-34b-3p	3.0204	2.9142	3.4609	3.0494	3.1930	3.3740	3.7356	4.8483
mir-34b-5p	-2.3537	-2.9728	-1.9847	-2.3335	-2.1199	-3.3402	-1.8035	-0.7809
mir-34c	0.0604	0.3482	1.0255	0.5681	0.7949	0.6796	1.0889	2.3604
mir-34c#	0.4024	0.6518	1.0848	0.5088	0.7610	0.9394	1.1247	2.1399
mir-350	-0.4232	-0.5990	-0.4400	-0.3823	-0.3449	-0.2771	-0.0565	-0.3690
mir-351	0.8608	NA	-0.5529	-1.1647	NA	NA	NA	NA
mir-361	-0.6894	-0.9978	-0.8087	-0.5102	-0.3100	-0.8488	-0.2189	-0.9292
mir-362-3p	-2.8250	-3.1374	-2.6418	-2.5249	-2.9295	-3.3122	-3.0951	-3.2208
mir-362-5p	-0.4147	-0.2367	-0.4814	-0.6349	-1.3624	-2.9815	-1.5753	-2.2525
mir-363	NA	NA	NA	NA	NA	NA	NA	NA
mir-365	0.5078	1.0526	1.0011	0.7276	1.2755	1.2027	1.9007	2.3305
mir-367	NA	NA	NA	NA	NA	NA	NA	NA
mir-369-3p	NA	NA	NA	NA	NA	NA	NA	NA
mir-369-5p	NA	NA	NA	NA	NA	NA	NA	NA
mir-370	NA	NA	NA	NA	NA	NA	NA	NA
mir-374-5p	3.3394	1.9942	2.5210	1.7247	1.6661	NA	1.5283	0.7194
mir-374#	NA	NA	NA	NA	NA	NA	NA	NA
mir-375	NA	NA	NA	NA	NA	NA	NA	-5.0359
mir-376a	NA	NA	NA	NA	NA	NA	NA	NA
mir-376a#	NA	NA	NA	NA	NA	NA	NA	NA
mir-376b	NA	NA	NA	NA	NA	NA	NA	NA
mir-376b#	-4.9517	NA	NA	NA	NA	NA	NA	-5.4922
mir-376c	-4.8870	NA	NA	NA	NA	NA	NA	NA
mir-376c#	NA	NA	NA	NA	NA	NA	NA	NA
mir-377	NA	NA	NA	NA	NA	NA	NA	NA
mir-378 (TM 2243)	-0.3520	-0.1564	-0.8256	-0.2947	-1.3967	-0.0564	-1.1613	-0.3601
mir-378 (TM 567)	-5.1070	NA	NA	NA	NA	NA	NA	NA
mir-379	-4.1193	NA	NA	NA	NA	NA	NA	NA
mir-380-3p	NA	NA	NA	NA	NA	NA	NA	NA
mir-380-5p	NA	NA	NA	NA	NA	NA	NA	NA
mir-381	NA	NA	NA	NA	NA	NA	NA	NA
mir-382	NA	NA	NA	NA	NA	NA	NA	NA
mir-382#	NA	NA	NA	NA	NA	NA	NA	NA
mir-383	-3.4263	NA	-4.1106	-5.3291	NA	NA	-5.2087	NA
mir-384-3p	-1.1309	-1.7951	-1.2940	-1.2803	-1.3030	-1.4101	-0.9551	-1.1880
mir-384-5p	3.4834	3.0172	3.1585	3.3231	3.5886	3.5790	3.8201	3.7034
mir-409-3p	-2.2519	NA	NA	NA	-3.7895	NA	-3.8185	NA
mir-409-5p	NA	NA	NA	NA	NA	NA	NA	NA
mir-410	NA	NA	NA	NA	NA	NA	NA	NA
mir-411	-4.3063	NA	-5.0913	NA	NA	NA	NA	NA
mir-411#	NA	NA	NA	NA	NA	NA	NA	NA
mir-412	NA	NA	NA	NA	NA	NA	NA	NA
mir-423-5p	-2.2291	-2.5158	-2.3155	-2.4116	-3.1015	-2.7672	-2.0453	-2.0373
mir-424	2.7520	0.6961	2.1844	1.8373	1.0333	0.9001	0.4412	0.5999
mir-425	-3.8965	-3.4949	-3.5327	-3.2601	-2.6407	-2.6143	-1.7964	-2.2834
mir-425-5p	NA	NA	NA	NA	NA	NA	NA	NA
mir-429	NA	NA	NA	NA	NA	NA	NA	NA
mir-431	NA	NA	NA	NA	NA	NA	NA	NA
mir-431#	NA	NA	NA	NA	NA	NA	NA	NA
mir-433	NA	NA	NA	NA	NA	NA	NA	NA
mir-433-5p	NA	NA	NA	NA	NA	NA	NA	NA
mir-434-3p	-4.1023	NA	NA	NA	NA	NA	-4.8362	NA
mir-434-5p	NA	NA	NA	NA	NA	NA	NA	NA
mir-448	NA	NA	NA	NA	NA	NA	NA	NA
mir-449 (TM 1030)	NA	-5.0681	NA	NA	NA	NA	NA	NA
mir-449b (TM 1667)	NA	NA	NA	NA	NA	NA	NA	NA
mir-449b (TM 2539)	NA	NA	NA	NA	NA	NA	NA	NA
mir-450a (TM 2303)	-2.9942	-3.8555	-2.8031	-3.4658	-3.5766	-4.0443	0.0796	-3.7334
mir-450a-3p	NA	NA	NA	NA	NA	NA	NA	NA
mir-450b	NA	NA	NA	NA	NA	NA	NA	NA
mir-452	NA	NA	NA	NA	NA	NA	NA	NA
mir-453	NA	NA	NA	NA	NA	NA	NA	NA
mir-455 (TM 1280)	-3.8070	-4.8608	-4.2959	-4.3960	-4.6009	NA	-3.9095	-5.4415

mir-455 (TM 2455)	-2.7572	-3.3165	-2.6951	-2.4059	-3.1265	-2.4398	-2.8019	-3.7233
mir-463#	NA	NA	NA	NA	NA	NA	NA	NA
mir-464	NA	NA	NA	NA	NA	NA	NA	NA
mir-465	NA	NA	NA	NA	NA	NA	NA	NA
mir-465a-3p	NA	NA	NA	NA	NA	NA	NA	NA
mir-465b-5p	NA	NA	NA	NA	NA	NA	NA	NA
mir-466a-3p	NA	NA	NA	NA	NA	NA	NA	NA
mir-466b-3-3p	NA	NA	NA	NA	NA	NA	NA	NA
mir-466c-5p	NA	NA	NA	NA	NA	NA	NA	NA
mir-466d-3p	NA	NA	NA	NA	NA	NA	NA	NA
mir-466d-5p	NA	NA	NA	NA	NA	NA	NA	NA
mir-466h	NA	NA	NA	NA	NA	NA	NA	NA
mir-467#	-3.7646	NA	NA	NA	NA	-3.9243	-4.0991	-3.9218
mir-467a (TM 1826)	0.1451	NA	0.2771	-0.1051	0.8804	1.1786	0.2401	-0.2709
mir-467a (TM 2587)	-3.6781	-2.4726	-2.8401	-2.0516	-2.5395	-1.6864	-2.0617	-1.1723
mir-467b	-2.7350	-2.9960	-3.0337	-2.9455	-2.5783	-3.0571	-2.8002	-3.0418
mir-467c	-4.3959	-3.1446	-4.1467	-3.7349	-4.1926	-3.4259	-4.0423	-3.1296
mir-467d	NA	NA	NA	NA	NA	NA	NA	NA
mir-467e	-5.0556	NA	NA	-4.5444	NA	-3.5831	-4.9117	-3.6530
mir-467e#	NA	NA	NA	NA	NA	NA	NA	NA
mir-468	NA	NA	NA	NA	NA	NA	NA	NA
mir-469	NA	NA	NA	NA	NA	NA	NA	NA
mir-470	NA	NA	NA	NA	NA	NA	NA	NA
mir-470#	NA	NA	NA	NA	NA	NA	NA	-5.5151
mir-471	NA	NA	NA	NA	NA	NA	NA	NA
mir-483#	NA	NA	NA	NA	NA	NA	NA	NA
mir-484	4.8780	4.4881	4.4434	4.3349	4.3125	3.8830	4.0063	3.9378
mir-485-3p	-3.3685	NA	NA	NA	NA	NA	NA	NA
mir-485-5p	NA	NA	NA	NA	NA	NA	NA	NA
mir-486	NA	NA	NA	NA	NA	NA	NA	NA
mir-487b	NA	NA	NA	NA	NA	NA	NA	NA
mir-488# (TM 1659)	-2.6065	-2.3570	-2.0831	-1.4927	-2.6881	-3.6670	-4.2377	-4.0130
mir-488# (TM 2014)	NA	NA	NA	NA	NA	NA	NA	NA
mir-489	NA	NA	NA	NA	NA	NA	NA	NA
mir-490	NA	NA	NA	NA	NA	NA	NA	NA
mir-491	-3.0511	-4.1288	-3.4701	-2.8440	-2.3707	-2.1433	-1.8775	-1.7155
mir-493	NA	NA	NA	NA	NA	NA	NA	NA
mir-494	-2.1835	-3.3757	-2.9612	-2.5198	-2.5259	-2.0072	-2.0171	-2.1104
mir-495	NA	NA	NA	NA	NA	NA	NA	NA
mir-496	NA	NA	NA	NA	NA	NA	NA	NA
mir-497	-0.8631	-2.1555	-1.1495	-0.8605	-1.0517	-1.2140	-1.1602	-0.3020
mir-499	NA	NA	NA	NA	NA	NA	NA	NA
mir-500	-3.6965	-4.6764	-4.1307	-3.3038	-4.4504	-5.5760	-3.9725	-5.2880
mir-501	NA	NA	NA	NA	NA	NA	NA	NA
mir-501#	NA	NA	NA	NA	NA	NA	NA	NA
mir-503	-1.3412	NA	-1.8498	-2.1664	NA	-3.5563	-3.7701	NA
mir-503#	-2.2553	-4.5139	-2.6317	-2.9574	-3.3191	-3.6482	-3.4466	-3.6483
mir-504	NA	NA	NA	NA	NA	NA	NA	NA
mir-505	NA	NA	NA	NA	NA	NA	NA	NA
mir-509-3p	NA	NA	NA	NA	NA	NA	NA	NA
mir-509-5p	NA	NA	NA	NA	NA	NA	NA	NA
mir-511	NA	NA	NA	NA	NA	NA	NA	NA
mir-532 (TM 1518)	2.7728	2.3044	2.5236	2.1555	1.9613	1.3089	1.4977	1.1115
mir-532-3p	1.7120	1.2396	1.5074	1.5050	0.7416	0.6773	0.5214	0.2334
mir-540	NA	NA	NA	NA	NA	NA	NA	NA
mir-540-5p	NA	NA	NA	NA	NA	NA	NA	NA
mir-541	-4.5305	NA	NA	NA	-5.1104	NA	-4.7442	NA
mir-542-3p	-5.1484	NA	-4.6166	-5.2942	NA	-5.4923	NA	NA
mir-542-5p	NA	NA	NA	NA	NA	NA	NA	NA
mir-543	NA	NA	NA	NA	NA	NA	NA	NA
mir-544	NA	NA	NA	NA	NA	NA	NA	NA
mir-546	NA	NA	NA	NA	NA	NA	NA	NA
mir-547	-2.5240	-4.6613	-3.0031	-3.5980	-2.9167	-4.0468	-3.5908	-4.3524
mir-551b	NA	NA	NA	NA	NA	NA	NA	NA
mir-574-3p	-2.7811	-2.1752	-2.6846	-1.6962	-1.1950	-0.5701	-0.4567	-0.3756
mir-582-3p	NA	NA	NA	NA	NA	NA	NA	NA
mir-582-5p	NA	NA	NA	NA	NA	NA	NA	NA
mir-590-5p	NA	NA	NA	NA	NA	NA	NA	NA
mir-592	NA	NA	NA	NA	-4.8400	NA	NA	NA
mir-598	-2.6430	-2.8878	-2.9707	-2.4462	-2.5108	-2.6260	-2.3016	-3.3674
mir-615	NA	NA	NA	NA	NA	NA	NA	NA
mir-615-5p	NA	NA	NA	NA	NA	NA	NA	NA
mir-652	1.2175	0.4929	1.1057	1.0718	1.2137	1.4712	1.2839	1.4600
mir-653	NA	NA	NA	NA	NA	NA	NA	NA
mir-654-3p	NA	NA	NA	NA	NA	NA	NA	NA
mir-654-5p	NA	NA	NA	NA	NA	NA	NA	NA
mir-665	NA	NA	NA	NA	NA	NA	NA	NA
mir-666	NA	NA	NA	NA	NA	NA	NA	NA
mir-666-3p	NA	NA	NA	NA	NA	NA	NA	NA
mir-667	-4.1463	-3.9874	-3.6210	-4.0873	-3.8679	-3.7135	-3.0713	-4.3584
mir-668	NA	NA	NA	NA	NA	NA	NA	NA
mir-669a	-3.4337	-3.2085	-3.2457	-3.4849	-3.6669	-2.5855	-2.9363	-2.6421
mir-670	NA	NA	NA	NA	NA	NA	NA	NA
mir-671-3p	-1.0255	-1.1410	-0.8147	-0.9611	-0.7433	-0.9911	-0.7245	-0.5310

mir-672	2.6407	2.4172	1.8997	1.2122	0.5744	-0.4653	0.2320	-0.0959
mir-673	-4.9036	NA	-5.3421	-5.0611	-4.3361	NA	-5.0055	NA
mir-673-3p	NA	NA	NA	NA	NA	NA	NA	NA
mir-674	-0.8272	-0.8003	-0.7670	-0.8517	-2.6814	-3.0819	-2.3654	NA
mir-674#	1.2677	1.0738	0.8957	0.7795	-0.4450	-1.4259	-0.5222	-0.9507
mir-675-3p	NA	NA	NA	NA	NA	NA	NA	NA
mir-675-5p	NA	NA	NA	NA	NA	NA	NA	NA
mir-676	-2.1414	-4.4730	-2.5179	-4.2371	-2.3267	-3.3551	-2.8955	-2.9801
mir-676#	NA	NA	NA	NA	NA	NA	NA	NA
mir-677	NA	NA	NA	NA	NA	NA	NA	NA
mir-678	-0.7864	-0.7374	-1.2410	-1.2909	-0.8581	-1.8517	-0.9989	-1.6296
mir-679	NA	NA	NA	NA	NA	NA	NA	NA
mir-680	NA	NA	NA	NA	NA	NA	NA	NA
mir-681	NA	NA	NA	NA	NA	NA	NA	NA
mir-682	NA	NA	-4.6842	NA	NA	NA	-4.5182	NA
mir-683	NA	NA	NA	NA	NA	NA	NA	NA
mir-684	NA	NA	NA	NA	NA	NA	NA	NA
mir-685	1.7844	-1.8384	2.0988	2.6407	0.6881	2.0479	2.7915	2.9533
mir-686	NA	NA	NA	NA	NA	NA	NA	NA
mir-687	NA	NA	NA	NA	NA	NA	NA	NA
mir-688	NA	NA	NA	NA	NA	NA	NA	NA
mir-690	3.9623	3.2054	4.3712	3.1280	3.3968	2.6184	3.3485	3.2277
mir-691	NA	NA	NA	NA	NA	NA	NA	NA
mir-692	NA	-3.3071	NA	NA	NA	-3.8439	-2.7215	-4.3942
mir-693	NA	NA	NA	NA	NA	NA	NA	NA
mir-693-3p	NA	NA	NA	NA	NA	NA	NA	NA
mir-694	NA	NA	NA	NA	NA	NA	NA	NA
mir-695	NA	NA	NA	NA	NA	NA	NA	NA
mir-696	NA	NA	NA	NA	NA	NA	NA	NA
mir-697	NA	NA	NA	NA	NA	NA	NA	NA
mir-698	NA	NA	NA	NA	NA	NA	NA	NA
mir-699	4.1929	3.6783	2.9225	2.9553	2.9991	1.7067	2.1790	2.6647
mir-7# (TM 1338)	0.2441	0.0446	-0.1645	-0.4482	-0.5548	-1.0607	-0.7503	-1.0383
mir-700	-1.0166	-1.1894	-1.2498	-1.3770	-0.3085	-1.2435	-0.3607	-0.4688
mir-701	NA	NA	NA	NA	NA	NA	NA	NA
mir-702	-4.7280	-4.1388	-5.0116	-4.9858	NA	NA	-4.2994	NA
mir-704	-4.5597	-4.4424	-4.4476	-3.8255	-4.4108	NA	-4.4201	-4.9193
mir-706	1.4018	-0.2429	0.7658	0.0980	1.0135	1.2927	0.3821	-0.6384
mir-707	NA	NA	NA	NA	NA	NA	NA	NA
mir-708	-2.2669	-2.4607	-2.0477	-0.2511	-2.2765	-2.5035	-2.0704	NA
mir-708#	NA	NA	NA	NA	NA	NA	NA	NA
mir-709	9.5762	7.8502	8.8088	7.9251	8.0902	8.3607	8.1183	7.6169
mir-710	NA	NA	NA	NA	NA	NA	NA	NA
mir-711	NA	NA	NA	NA	NA	NA	NA	NA
mir-712#	NA	NA	NA	NA	NA	NA	NA	NA
mir-713	NA	NA	NA	NA	NA	NA	NA	NA
mir-715	-1.9953	NA	NA	-2.6344	NA	NA	NA	NA
mir-717	NA	NA	NA	NA	NA	NA	NA	NA
mir-718	NA	NA	NA	NA	NA	NA	NA	NA
mir-719	NA	NA	NA	NA	NA	NA	NA	NA
mir-720	5.1959	4.1480	6.0204	4.9177	5.4897	4.5028	5.6999	5.5078
mir-721	-3.9188	-3.0154	-3.9433	-3.4681	-2.9881	-4.9619	-3.5040	-3.7872
mir-741	NA	NA	NA	NA	NA	NA	NA	NA
mir-742	NA	NA	NA	NA	NA	NA	NA	NA
mir-742#	NA	NA	NA	NA	NA	NA	NA	NA
mir-743a	NA	NA	NA	NA	NA	NA	NA	NA
mir-743b-3p	NA	NA	NA	NA	NA	NA	NA	NA
mir-743b-5p	NA	NA	NA	NA	NA	NA	NA	NA
mir-744	2.1249	1.7537	1.7414	1.9772	1.9868	1.5034	1.9854	1.6101
mir-744#	-5.0229	-4.8383	-4.0739	NA	-3.8734	NA	-3.4125	-3.0474
mir-758	NA	NA	NA	NA	NA	NA	NA	NA
mir-759	NA	NA	NA	NA	NA	NA	NA	NA
mir-760	0.9263	0.9733	1.1755	1.0780	1.4140	0.0965	1.2812	1.1986
mir-761	NA	NA	NA	NA	NA	NA	NA	NA
mir-762	NA	NA	NA	NA	NA	NA	NA	NA
mir-763	NA	NA	NA	NA	NA	NA	NA	NA
mir-764-3p	NA	NA	NA	NA	NA	NA	NA	NA
mir-764-5p	-2.0166	-2.1336	-2.3104	-2.3335	-1.9357	-2.8633	-2.5763	-2.7646
mir-770-3p	NA	NA	NA	NA	NA	NA	NA	NA
mir-770-5p	NA	NA	NA	NA	NA	NA	NA	NA
mir-7b	NA	NA	NA	NA	NA	NA	NA	NA
mir-802	NA	NA	NA	NA	NA	NA	NA	NA
mir-804	-4.7076	NA	-4.8489	-5.3310	-4.9806	NA	-4.0563	NA
mir-805	3.7272	3.2236	3.0631	2.4926	2.6922	1.9519	2.3769	1.9407
mir-871	NA	NA	NA	NA	NA	NA	NA	NA
mir-872	0.6090	0.1209	0.5940	0.7260	1.1810	1.4612	1.2590	1.9784
mir-872#	-0.7165	-0.8145	-0.2342	-0.7714	0.1657	0.2021	0.1940	0.8258
mir-873	NA	NA	NA	NA	-4.7304	NA	-4.4479	NA
mir-874	NA	NA	NA	NA	NA	NA	NA	NA
mir-875-3p	NA	NA	NA	NA	NA	NA	NA	NA
mir-875-5p	-4.8531	-4.7484	NA	NA	-4.9489	NA	-4.6531	-4.9902
mir-876-3p	NA	NA	NA	NA	NA	NA	NA	NA
mir-876-5p	NA	NA	NA	NA	NA	NA	NA	NA
mir-877#	0.6570	0.2544	0.3301	0.3546	0.4753	-0.9592	0.4807	-0.2076

mir-878-3p	NA	NA	NA	NA	NA	NA	NA	NA
mir-878-5p	NA	NA	NA	NA	NA	NA	NA	NA
mir-879	NA	NA	NA	NA	NA	NA	NA	NA
mir-879#	-4.0446	-2.6964	-4.1568	-4.1687	-2.6326	NA	-2.6214	-4.2512
mir-881	NA	NA	NA	NA	NA	NA	NA	NA
mir-881#	NA	NA	NA	NA	NA	NA	NA	NA
mir-882	NA	NA	NA	NA	NA	NA	NA	NA
mir-883a-3p	NA	NA	NA	NA	NA	NA	NA	NA
mir-883a-5p	NA	NA	NA	NA	NA	NA	NA	NA
mir-883b-3p	NA	NA	NA	NA	NA	NA	NA	NA
mir-9	7.5172	8.9125	9.1666	9.5082	8.3759	8.7724	8.1006	8.4017
mir-9#	8.5054	7.6427	8.6136	8.5643	7.7714	6.5883	7.4499	7.1219
mir-92	5.8914	5.2288	4.9253	4.6440	4.6353	3.4288	4.8479	2.9480
mir-92a#	NA	NA	NA	NA	NA	NA	NA	NA
mir-93	4.0013	3.7725	3.7366	3.5355	2.7182	2.7393	2.5775	2.4966
mir-93#	2.2092	1.7097	1.8258	1.6241	1.0097	0.9070	0.6950	0.7789
mir-96	NA	NA	NA	NA	NA	NA	NA	NA
mir-98	NA	NA	NA	NA	NA	NA	NA	NA
mir-99a	3.9393	3.3094	3.9789	4.0969	4.3548	4.5094	4.2922	4.7732
mir-99b	2.7554	2.8751	2.6090	2.5011	2.8619	3.3243	2.8401	3.2876
mir-99b#	-1.8910	-2.2782	-2.5707	-2.8356	-2.3216	-2.1314	-2.3066	-2.5027
SNORNA135	6.2636	5.3984	5.1263	5.7896	5.3090	4.5543	4.9848	4.8052
SNORNA234	NA	NA	NA	NA	NA	NA	NA	NA
SNORNA251	NA	NA	NA	NA	NA	NA	NA	NA
SNORNA292	NA	NA	NA	NA	NA	NA	NA	NA
SNORNA412	NA	NA	NA	NA	NA	NA	NA	NA
SNORNA420	NA	NA	NA	NA	NA	NA	NA	NA
snoRNA429	9.8287	8.9900	9.1990	9.6321	9.4923	9.1222	NA	9.1053
snoRNA429	7.3839	6.1530	6.3996	6.6133	6.4169	6.4563	-1.3949	6.1087
SNORNA429	7.2539	6.2125	5.9689	6.7292	6.5369	6.5161	6.1851	6.3404
SNORNA55	NA	NA	NA	NA	NA	NA	NA	NA
snoRNRA135	6.0623	5.4641	5.2696	5.6826	5.5292	4.7610	NA	4.6394
U6	8.8579	8.2694	7.8922	7.7098	7.8929	7.2120	6.0584	7.2420
