# Supplementary material for: Dynamic Activity of miR-125b and miR-93 during Murine Neural Stem Cell Differentiation In Vitro and in the Subventricular Zone Neurogenic Niche
Source: PLoS One. 2013 Jun 27;8(6):e67411. doi: 10.1371/journal.pone.0067411 (PMC3694868; doi:10.1371/journal.pone.0067411)
Supplement: Table S2 — Heatmap of the most variable top-ranked miRNAs. Heatmap showing the list of miRNAs that are modulated along the differentiation process. Data are expressed as ΔCt normalized on mean expression value. We assigned an arbitrary color code referring to the relative abundance of each miRNA. We reported miRNAs that displayed differential expression (ΔΔCt ≥1) in progenitors and/or differentiated cells as compared to stem/precursors. #1 and #2 indicate two independent NSC lines. (PDF) [file pone.0067411.s004.pdf]

Supplementary Table 2. Heatmap of the most variable top-ranked miRNAs.

Heatmap showing shortlisted miRNAs that are moduated along the differentiation process. Data are expressed as **Δ**Ct normalized on mean expression value. The arbitrary color code refers to the relative abundance of each miRNA. We reported miRNAs that displayed differential expression (**ΔΔ**Ct ≥ 1) in progenitors and/or differentiated cells (7 and 10 days of differentiation) as compared to stem/precursor cells. #1 and #2 indicate two independent NSC lines.

	**Stem/precursors**		**Progenitors**		**Differentiated cells**			
					**(7d)**		**(10d)**	
	#1	#2	#1	#2	#1	#2	#1	#2
mir-125b (TM 449)	5.87	5.24	6.10	6.51	6.47	6.55	6.31	6.79
mir-92	5.89	5.23	4.93	4.64	4.64	3.43	4.85	2.95
mir-132	4.59	2.04	4.49	2.02	1.65	1.98	2.61	2.61
mir-93	4.00	3.77	3.74	3.54	2.72	2.74	2.58	2.50
mir-139-5p	4.08	3.38	3.10	2.48	0.74	1.11	-0.32	0.43
mir-335 (TM 546)	3.37	3.46	3.63	3.38	2.20	1.78	1.74	1.69
mir-532 (TM 1518)	2.77	2.30	2.52	2.16	1.96	1.31	1.50	1.11
mir-20b	2.79	2.13	2.28	1.46	2.31	0.52	1.89	-0.49
mir-129-3p	2.07	2.31	1.03	0.36	0.72	0.41	0.32	0.89
mir-212	2.51	2.69	-0.08	0.03	-0.36	0.89	0.33	0.77
mir-672	2.64	2.42	1.90	1.21	0.57	-0.47	0.23	-0.10
mir-374-5p	3.34	1.99	2.52	1.72	1.67		1.53	0.72
mir-424	2.75	0.70	2.18	1.84	1.03	0.90	0.44	0.60
mir-146a	0.57	2.07	1.14	2.48	-0.15	1.26	-0.50	-1.49
mir-335#	1.70	2.45	1.44	0.65	-0.61	-0.82	-0.71	-1.20
mir-93#	2.21	1.71	1.83	1.62	1.01	0.91	0.69	0.78
mir-532-3p	1.71	1.24	1.51	1.50	0.74	0.68	0.52	0.23
mir-25	1.97	1.09	1.07	0.66	0.39	0.59	0.84	0.50
mir-29b#	1.73	1.95	-0.90	-1.65	-1.19	-2.08	-0.90	-1.39
mir-30a-5p	1.57	1.48	1.71	1.82	2.16	2.35	2.73	2.87
mir-674#	1.27	1.07	0.90	0.78	-0.45	-1.43	-0.52	-0.95
mir-138	1.03	-0.40	1.39	0.04	5.60	0.45	6.01	2.18
mir-872	0.61	0.12	0.59	0.73	1.18	1.46	1.26	1.98
mir-146b	0.51	0.61	0.63	0.66	1.52	1.79	1.65	1.39
mir-27a	-0.73	0.61	-0.48	0.28	0.78	2.00	1.33	2.15
mir-27b	0.07	0.11	-0.01	0.13	1.67	1.74	2.26	1.82
mir-140	0.73	0.14	0.79	0.93	1.88	2.74	1.89	3.28
mir-22	-1.32	0.29	0.07	0.70	1.70	1.27	2.20	2.69
mir-152	-1.97	-1.94	-1.38	-0.45	0.82	0.99	1.03	1.78
mir-24-2#	-2.31	-1.13	-2.52	-2.12	-0.60	-0.25	-0.36	0.68
mir-145	-4.78	-3.77	-3.79	-1.64	-1.57	-0.43	-1.10	-0.31

**Heatmap**						
	ΔCt >4	4 <ΔCt <2	2 <ΔCt < 1	1 <ΔCt < -1	4 < ΔCt < -2	ΔCt < -2
