# Supplementary material for: Dynamic Activity of miR-125b and miR-93 during Murine Neural Stem Cell Differentiation In Vitro and in the Subventricular Zone Neurogenic Niche
Source: PLoS One. 2013 Jun 27;8(6):e67411. doi: 10.1371/journal.pone.0067411 (PMC3694868; doi:10.1371/journal.pone.0067411)
Supplement: Methods S1 — (DOCX) [file pone.0067411.s005.docx]

**Supplementary Information**

**Supplementary methods**

**List of oligonucleotides used to generate transfer vector plasmids**

miR-125b sense 1: ctagatcacaagttagggtctcagggacgattcacaagttagggtctcagggaacgcgt

miR-125b sense 2: tcacaagttagggtctcagggatcactcacaagttagggtctcagggac

miR-125b antisense 1: tccctgagaccctaacttgtgaatcgtccctgagaccctaacttgtgat

miR-125b antisense 2: ccgggtccctgagaccctaacttgtgagtgatccctgagaccctaacttgtgaacgcgt

miR-124 sense 1: ctagataatggcattcaccgcgtgccttaattcgaatggcattcaccgcgtgccttaaacgcgt

miR-124 sense 2: tggcattcaccgcgtgccttaaatgcattggcattcaccgcgtgccttaac

miR-124 antisense 1: ttaaggcacgcggtgaatgccattcgaattaaggcacgcggtgaatgccattat

miR-124 antisense 2: ccgggttaaggcacgcggtgaatgccaatgcatttaaggcacgcggtgaatgccaacgcgt

miR-93-5p sense1: ctagactacctgcacgaacagcactttgttcgaactacctgcacgaacagcactttgacgcgt

miR-93-5p sense2: ctacctgcacgaacagcactttgatgcatctacctgcacgaacagcactttgc

miR-93-5p antisense1: caaagtgctgttcgtgcaggtagttcgaacaaagtgctgttcgtgcaggtagt

miR-93-5p antisense2: ccgggcaaagtgctgttcgtgcaggtagatgcatcaaagtgctgttcgtgcaggtagacgcg

miR-23a sense: ctagatagggaaatccctggcaatgtgatcgatggaaatccctggcaatgtgatc

miR-23a antisense: ccgggatcacattgccagggatttccatcgatcacattgccagggatttccctat

NB: 4 copies of miR-23a were generated by successive ligation of 2 oligonucleotide products (each containing 2 tandem repeats complementary to miR-23a) into the pBlueNA subcloning construct.

**Vector production and titration**

VSV-pseudotyped third-generation LV were produced by transient four-plasmid co-transfection into 293T cells and purified by ultracentrifugation as described [1]. Vector titer was tested on 293T cells by limiting dilution and estimated by means of qPCR for HIV genome copies. Vector particles were measured by HIV-1 gag p24 antigen immunocapture (NEN Life Science Products, Waltham, MA, USA). Vector infectivity was calculated as the ratio between titer and particle. Details can be found in [1]. Vector titers ranged from 2-5x10^9^ TU/ml (bdLVs) and 5-8x10^9^ TU/ml (monocystronic LVs) with infectivity >5x10^4^ TU/ng of p24 for all vectors.

**Establishment of NSC-derived populations**

Serially passaged neurospheres were dissociated and grown for 24 hours in serum-free DMEM/F12 (1:1 vol:vol) containing insulin, apo-transferrin, putrescine and progesterone (*control medium*) containing FGF2 and EGF (*growth medium*), in order to obtain a population enriched in proliferating, undifferentiated cells (*stem/precursors; 1day, d*). This population was plated in the presence of an adhesion substrate, in control medium supplemented with FGF2 in order to obtain a population enriched in neuronal and glial committed progenitors at different stages of commitment (*progenitors; 3d*). Progenitors were exposed to control medium containing 10ng/ml leukaemia inhibitory factor (LIF) or 2% FBS and grown for additional 1d, 4d and 7d (*differentiated cells*) in order to achieve cultures at progressive stages of neuronal and glial differentiation/maturation. We used nestin to identify stem/precursor cells and immature glial precursor cells, glial fibrillary protein (GFAP) to identify astrocytes, β-tubulin III (βtubIII) to identify immature neurons, microtubule-associated protein 2 (Map2) and neuronal nuclear antigen (NeuN) to identify mature neurons (see also Figure S1**).**

**Primary and secondary antibodies**

Primary antibodies

Rabbit polyclonal anti-DS red (mCherry) (Clontech, Mountain View, CA, USA; 1:500); mouse monoclonal anti-β-tubulin III (Babco, Richmond, CA, USA; 1:1.000); rabbit polyclonal anti-β-tubulin III (Babco; 1:500); mouse monoclonal anti-GFAP (Chemicon-Millipore, Temecula, CA, USA; 1:1.000); rabbit polyclonal anti-BLBP (Chemicon; 1:1.000); rabbit polyclonal anti-GFAP (Dako, Glostrup, Denmark; 1:1.000); mouse monoclonal anti-Map2 (Immunological Science, Rome, Italy; 1:300); mouse monoclonal anti-nestin (Chemicon; 1:200); mouse monoclonal anti-NeuN (Chemicon; 1:500); rabbit polyclonal anti-Ki67 (Novocastra-Leica Biosystems GmbH, Nussloch, Germany; 1:1.000); chicken polyclonal anti-GFP (Abcam, Cambridge, UK; 1:1.000).

Secondary antibodies

Alexa 488-, Alexa 546- or Alexa 633-conjugated anti-mouse or anti rabbit IgG (1:1.000, 1:2.000 and 1:500, respectively) (Molecular Probes; Carlsbad, CA, USA); Cy3- conjugated goat anti-mouse or goat anti-rabbit IgG (Jackson ImmunoResearch Laboratories, West Grove, PA, USA; 1:500).

Coverslips and tissue sections were counterstained with dapi (4', 6-diamidino-2-phenylindole; Roche) or TO-PRO-3 (Life Technologies-Invitrogen, Carlsbad, CA, USA), washed in PBS, and mounted on glass slides using Fluorsave (Calbiochem-EMD Millipore, Billerica, MA, USA).

**Image acquisition**

Samples were visualized with: 1) Zeiss Axioskop2 microscope using double laser confocal microscopy with Zeiss Plan-Neofluar objective lens (Zeiss, Arese, Italy). Images were acquired using a Radiance 2100 camera (Bio-Rad, Segrate, Italy) and LaserSharp 2000 acquisition software (Bio-Rad); 2) Perkin Elmer UltraVIEW ERS Spinning Disk Confocal (PerkinElmer Life Sciences Inc., MA, USA); 3) Leica TCS SP2 three-laser confocal microscope with Leica Confocal Software (LCS; Leica Microsystems GmbH, Wetzlar, Germany).

**Detection of LV genome**

Genomic DNA was extracted from cell pellets (Maxwell, Promega, Madison, WI, USA) and quantified at NanoDrop ND-1000 Spectrophotometer (Euroclone, Pero, Italy). Vector copies per genome were quantified by TaqMan analysis starting from 100 ng of template DNA. Quantitative PCR was performed by amplifying the PSI sequence of the LV backbone using primers as follows: forward, 5′-TGAAAGCGAAAGGGAAACCA-3′, and reverse, 5′-CCGTGCGCGCTTCAG-3′. PCR product length was 64 bp at a final concentration of 750 nmol for forward and 200 nmol for reverse primers. The probe was 5′-VIC-AGCTCTCTCGACGCAGGACTCGGC- MGB-3′ at a 200 nmol final concentration. As internal reference for normalization, we amplified a fragment of the murine β-actin gene: forward, 5′-AGAGGGAAATCGTGCGTGAC-3′, 300 nmol final concentration; reverse, 5′-CAATAGTGATGACCTGGCCGT-3′, 750 nmol final concentration; probe, 5′-VIC-CACTGCCGCATCCTCTTCCTCCCMGB-3′, 200 nmol final concentration.

A standard curve of genomic DNA carrying 4 LV copies, validated by Southern blot analysis, was constructed using DNA extracted from transgenic mouse tissue. The standard curve, based on different dilutions of DNA (from 200 to 25 ng), and accordingly, of LV copies, was used as standard both for LVs and for β-actin amplification. Reactions were carried out in a total volume of 25 μl, in an ABI Prism 7900 HT Sequence Detection System (Life Technologies-Applied Biosystems, Carlsbad, CA, USA). The number of LV copies was calculated as follows: (ng LVs/ng endogenous DNA) × (number of LV integrations in the standard curve).

**Legends to Supplementary Figures**

**Supplementary Figure 1. BdLV-transduced NSCs maintain self-renewal ability and multipotency.**

**(A)** Cartoon summarizing the NSC culture system and the differentiation protocol. **(B)** Representative images showing neuronal (Map2, red) and glial progeny (GFAP, red) in NSC-derived populations during differentiation. Nuclei counterstained with DAPI (blue). Scale bar, 100 µm. **(C)** Cell counts performed after immunofluorescence analysis using lineage-specific markers showed similar cell type composition of untransduced (UT) and bdLV-transduced NSCs (bdLVs) at different stages of lineage commitment and differentiation. Data are mean ±SEM, n=5 independent experiment, 3 independent NSC cultures, 2-4 coverlips/experiment/antigen (data from bdLV.CTRL- and bdLV.miRT-transduced cells were pooled). **(D)** Clonogenic efficiency and **(E)** long-term proliferation ability of NSCs are not altered following transduction with bdLVs. Data in (D) are the mean±SEM, n=5 independent experiments (data from bdLV.CTRL- and bdLV.miRT-transduced cells were pooled) Data in (E) are the mean±SEM, n= 3 NSC independent cultures (data from bdLV.CTRL- and bdLV.miRT-transduced cells were pooled). NSCs were analyzed starting from 6 passages after transduction (total subculturing passages between 12 and 16).

**Supplementary Figure 2. Activity of miR-125b and miR-93 in proliferating precursor and progenitor cell populations.**

**(A)** Integrated LV genome (vector copy number, VCN) measured by qPCR in LV.CTRL-, LV.miRT125b- and LV.miRT93-transduced *stem/precursors.* Transduction efficiency (expressed as percentage of GFP^+^ cells assessed by indirect IF analysis) was 80.53 ± 1.1 in LV.CTRL-transduced cells. LV.miRT-transduced cells show VCN that are comparable or higher than LV.CTRL-transduced cells, suggesting comparable or even higher transduction efficiency. Data are expressed as mean ± SD, n=2 independent NSC lines. **(B)** Quantitative analysis of GFP expression in Ki67^+^nestin^+^ cells (on total Ki67^+^) and Ki67^-^nestin^+^ cells (on total nestin^+^) in LV.CTRL-, LV.miRT125b- and LV.miRT93-transduced *stem/precursors*. GFP^-^ cells in LV.CTRL-transduced cultures represent untranduced cells. The GFP^-^ cell population in LV.miRT-transduced *stem/precursors* is composed by a small percentage of untransduced cells while in the remaining cells GFP expression is low/absent due to the high activity of the endogenous miRNA. The proportion of GFP^+^ cells is significantly decreased in the nestin^+^Ki67^+^ cell population but not in the nestin^+^Ki67^-^ cell population as compared to LV.CTRL-transduced cells, revealing high activity of miR-125b and miR-93 in cycling precursors. Data are the mean ± SEM; n=2 experiments, 2 NSC lines/experiment. Data were analyzed by one-way analysis of variance followed by Bonferroni’s posttest. *p<0.01 versus LV.CTRL-transduced cells. (**C**) Representative images of LV.CTRL-, LV.miRT125b- and LV.miRT93-transduced *stem/precursors* showing GFP expression in Ki67^+^Nestin^+^ cells (arrows). Arrowheads identify Ki67^+^Nestin^+^GFP^-^ cells. Scale bars, 100 µm.

**Supplementary Table 1. miRNA expression profile in NSCs and differentiated progeny.**

In order to identify novel miRNA candidates enriched and/or highly modulated in NSC-derived populations along the differentiation stages, we performed a high-throughput miRNA RT-qPCR in a time course differentiation analysis considering *stem/precursors*, *committed progenitors* and *differentiated cells* at two different stages (7d and 10d in vitro; see Figure S1). A total of 535 mammalian miRNAs were interrogated. Among them, 201 displayed detectable expression level (Ct ≤ 32). We used the mean expression value in a given sample to normalize high-throughput miRNA RT-qPCR data [2], [3]. Levels of miRNA expression are expressed as ΔCt.

**Supplementary Table 2. Heatmap of the most variable top-ranked miRNAs.**

Heatmap showing the list of miRNAs that are modulated along the differentiation process.

Data are expressed as ∆Ct normalized on mean expression value. We assigned an arbitrary color code referring to the relative abundance of each miRNA. We reported miRNAs that displayed differential expression (∆∆Ct ≥ 1) in *progenitors* and/or *differentiated cells* as compared to *stem/precursor cells*. #1 and #2 indicate two independent NSC lines.

**References**

1. Amendola M, Venneri MA, Biffi A, Vigna E, Naldini L (2005) Coordinate dual-gene transgenesis by lentiviral vectors carrying synthetic bidirectional promoters. Nat Biotechnol 23: 108-116.

2. Mestdagh P, Bostrom AK, Impens F, Fredlund E, Van Peer G, et al. (2010) The miR-17-92 microRNA cluster regulates multiple components of the TGF-beta pathway in neuroblastoma. Mol Cell 40: 762-773.

3. Mestdagh P, Feys T, Bernard N, Guenther S, Chen C, et al. (2008) High-throughput stem-loop RT-qPCR miRNA expression profiling using minute amounts of input RNA. Nucleic Acids Res 36: e143.
